# KLF6 alleviates hepatic ischemia-reperfusion injury by inhibiting autophagy

**DOI:** 10.1038/s41419-023-05872-3

**Published:** 2023-07-01

**Authors:** Jiye Li, Dongsheng Yu, Chenhui He, Qiwen Yu, Zhongkun Huo, Yi Zhang, Shuijun Zhang

**Affiliations:** 1 Department of Hepatobiliary and Pancreatic Surgery, The First Affiliated Hospital of Zhengzhou University, Zhengzhou, Henan China; 2 Henan Key Laboratory for Digestive Organ Transplantation, The First Affiliated Hospital of Zhengzhou University, Zhengzhou, Henan China; 3 Department of Emergency, The First Affiliated Hospital of Zhengzhou University, Zhengzhou, Henan China; 4 Department of Anesthesiology, Pain and Perioperative Medicine, The First Affiliated Hospital of Zhengzhou University, Zhengzhou, Henan China; 5 Department of Orthopaedic Surgery, The First Affiliated Hospital of Zhengzhou University, Zhengzhou, Henan China

**Keywords:** Liver diseases, Predictive markers

## Abstract

Hepatic ischemia-reperfusion (I/R) injury, a common clinical complication of liver transplantation, gravely affects patient prognosis. Krüppel-like factors (KLFs) constitute a family of C2/H2 zinc finger DNA-binding proteins. KLF6, a member of the KLF protein family, plays crucial roles in proliferation, metabolism, inflammation, and injury responses; however, its role in HIR is largely remains unknown. After I/R injury, we found that KLF6 expression in mice and hepatocytes was significantly upregulated. Mice were then subjected to I/R following injection of shKLF6- and KLF6-overexpressing adenovirus through the tail vein. KLF6 deficiency markedly exacerbated liver damage, cell apoptosis, and activation of hepatic inflammatory responses, whereas hepatic overexpression of KLF6 in mice produced the opposite results. In addition, we knocked out or overexpressed KLF6 in AML12 cells before exposing them to a hypoxia-reoxygenation challenge. KLF6 knockout decreased cell viability and increased hepatocyte inflammation, apoptosis, and ROS, whereas KLF6 overexpression had the opposite effects. Mechanistically, KLF6 inhibited the overactivation of autophagy at the initial stage, and the regulatory effect of KLF6 on I/R injury was autophagy-dependent. CHIP-qPCR and luciferase reporter gene assays confirmed that KLF6 bound to the promoter region of Beclin1 and inhibited its transcription. Additionally, KLF6 activated the mTOR/ULK1 pathway. Finally, we performed a retrospective analysis of the clinical data of liver transplantation patients and identified significant associations between KLF6 expression and liver function following liver transplantation. In conclusion, KLF6 inhibited the overactivation of autophagy via transcriptional regulation of Beclin1 and activation of the mTOR/ULK1 pathway, thereby protecting the liver from I/R injury. KLF6 is expected to serve as a biomarker for estimating the severity of I/R injury following liver transplantation.

## Introduction

Liver transplantation (LT) is an effective strategy for the treatment of liver cancer and end-stage liver diseases [1, 2]. However, from the harvesting and cold preservation of the donor organs to the anastomosis of the donor and recipient vessels and the restoration of hepatic blood flow, ischemia-reperfusion (I/R) injury is inevitable. This often results in impaired graft function, including early allograft dysfunction, primary nonfunction, ischemic bile duct injury, and recurrence of liver cancer after transplantation [3–5]. Currently, the primary methods to alleviate I/R injury include ischemic preconditioning, drug application, and machine perfusion [6, 7]. However, due to their limitations, the popularization and clinical application of these techniques have been challenging [8, 9]. Therefore, it is necessary to elucidate the mechanism underlying the occurrence and development of I/R injury in order to develop novel targets and methods for clinical treatment.

The family of Krüppel-like factors (KLFs) regulates multiple cellular biological processes, including proliferation, differentiation, metabolism, as well as the maintenance of cellular pluripotency, inflammation, and injury responses [10]. KLF6, a member of the KLF family, is a widely expressed nuclear transcriptional regulator [11]. It regulates the transcription of PDGFB to activate the mTOR signaling pathway, thereby regulating lipid metabolism to promote the progression of renal clear cell carcinoma [12]. Podocyte-specific deletion of KLF6 increases the mitochondrial damage and apoptosis in diabetic nephropathy [13]. However, whether and how KLF6 plays a role in hepatic I/R injury remains unknown.

Autophagy is a conserved intracellular catabolic process that delivers cellular components to the lysosome for degradation, providing nutrients and the necessary material basis for cell survival, thereby playing pivotal roles in maintaining cellular homeostasis [14]. Accumulating evidence suggests that autophagy plays crucial role in I/R injury of the heart, liver, brain, kidney, and lungs [15]. As a compensatory mechanism, autophagy alleviates the detrimental effects of ATP deprivation on cells during ischemia, thereby exerting a protective effect. However, during reperfusion, persistent and excessive activation of autophagy can lead to cell death. Multiple studies on I/R injury on the heart, liver, and brain corroborate this viewpoint [16]. The regulation of autophagy is significantly influenced by the KLF family. Conserved KLFs/autophagy pathways regulate nematode lifespan and age-related vascular deterioration in mammals [17]. The interaction between autophagy and KLF2 in acute liver injury determines the endothelial phenotype and microvascular function [18]. However, the regulatory effect and underlying mechanism of KLF6 on autophagy in hepatic I/R injury are unknown. The current study, therefore, aimed to: (1) assess the expression of KLF6 in hepatic I/R injury; (2) elucidate the regulatory effects of KLF6 on hepatic I/R injury; (3) confirm whether KLF6 alleviated hypoxia-reoxygenation (H/R) injury in AML12 cells by inhibiting the overactivation of autophagy and, if so, its underlying molecular mechanism; and (4) assess the correlation between KLF6 expression and liver function after clinical LT.

## Results

### Increased KLF6 expression is associated with hepatic I/R injury

**Fig. 1:**
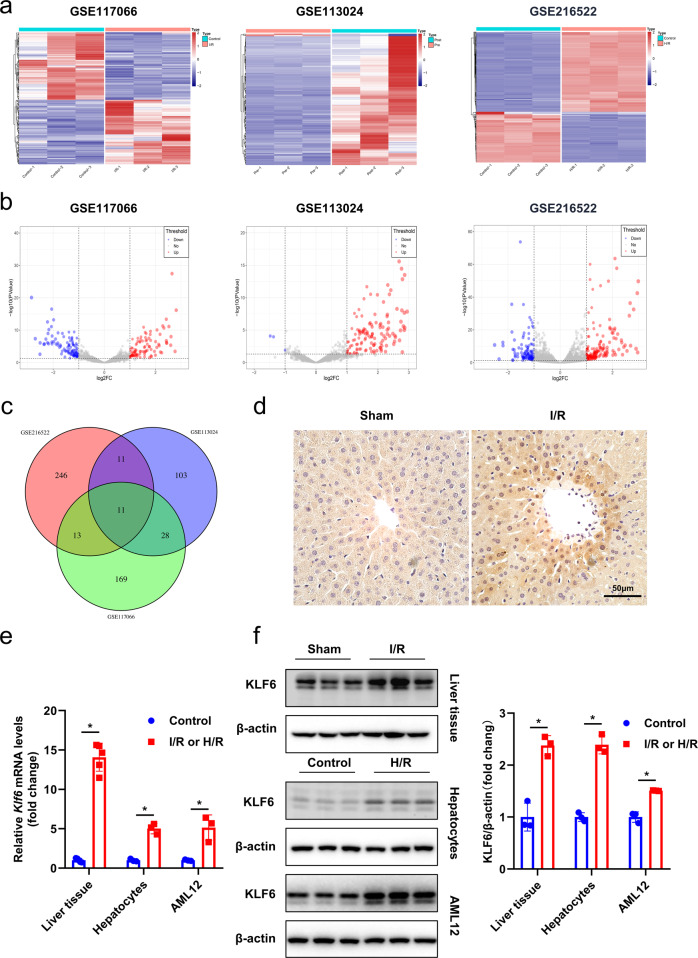
Hepatic I/R injury induces the upregulation of KLF6. **a**, **b** Heatmap and volcano plot for differentially expressed genes identified in GSE113024, GSE117066 and our RNA-Seq dataset. **c** Venn diagram of the number of co- and differentially expressed genes among the three datasets. **d** Liver tissues were immunohistochemically stained for KLF6 after ischemia/reperfusion (*n* = 5 per group). Scale bar, 50 μm. **e**, **f** KLF6 mRNA and protein levels were detected with RT-qPCR and western blotting analysis after I/R (ischemia 1 h and reperfusion 6 h) (*n* = 5 per group) or H/R (hypoxia 6 h and reoxygenation 2 h). Mean values ± SD are shown, and statistical significance was determined by two-tailed Student’s *t* test. *p < 0.05.

Bioinformatics-based approaches were used to identify the genes whose expression changed significantly after liver I/R injury. Two publicly available RNA-seq datasets (GSE117066 (mouse I/R), GSE113024 (human I/R)) and our RNA-seq dataset (GSE216522) from AML12 cells that were exposed to hypoxia for 6 h and reoxygenation for 2 h were used to identify genes of interest. The heatmap and volcano plot of the three datasets are shown in Fig. 1a, b. In addition, a Venn diagram was generated for the datasets to confirm the differential expression of the genes that are shared among mouse, human, and liver cell lines (Fig. 1c). Of these, *KLF6* was discovered among the final 11 genes examined. Immunohistochemical analysis of the mouse liver section after I/R injury revealed a significant upregulation of KLF6 expression (Fig. 1d) and its localization in both the nucleus and cytoplasm (predominantly in the nucleus). We also evaluated the mRNA and protein expression of KLF6 in liver tissues, primary hepatocytes, and AML12 cells and observed significant upregulation following I/R or H/R (Fig. 1e, f). Moreover, the immunofluorescence experiments on AML12 cells led to the same conclusion (Fig. S1).

### KLF6 deficiency exacerbates hepatic I/R injury

**Fig. 2:**
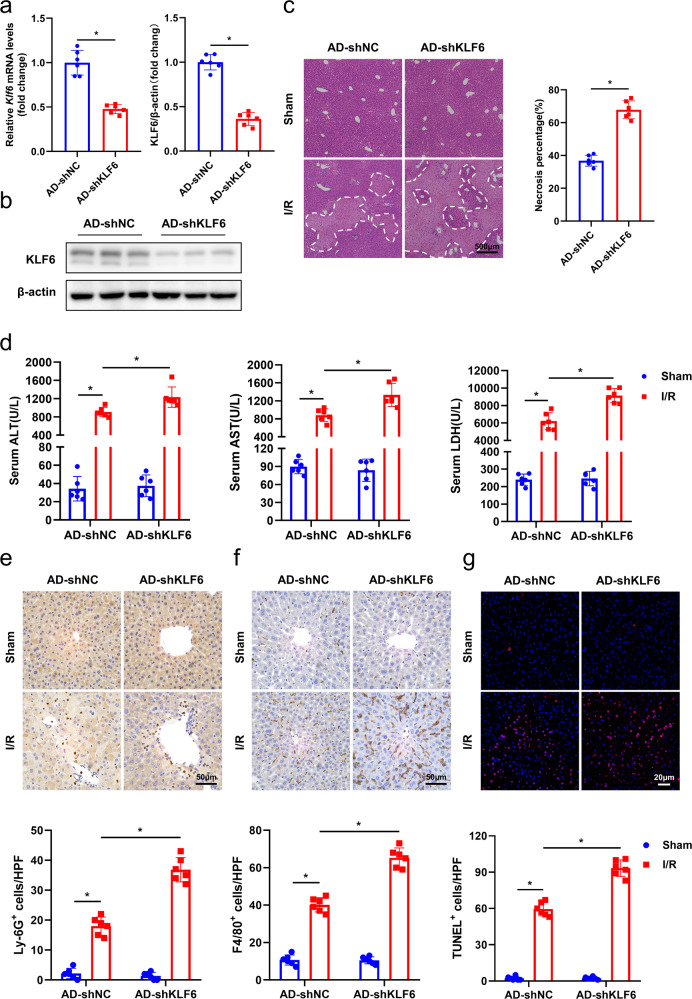
KLF6 knockdown exacerbates hepatocellular injury and inflammation during hepatic I/R. **a**, **b** Male C57BL/6N mice at 6–8 weeks of age were infected with KLF6 shRNA adenovirus via tail-vein injection. Seven days after the adenovirus injection, KLF6 expression was assessed by RT-qPCR and western blotting analysis. **c** Representative histological H&E-stained images showing necrotic areas in liver tissue from AD-shNC and AD-shKLF6 mice after hepatic I/R. Scale bar, 500 μm. **d** Serum ALT/AST/LDH levels of AD-shNC and AD-shKLF6 mice after hepatic I/R. **e**, **f** Representative pictures of liver stained with Ly-6G (neutrophils) and F4/80 (macrophages). Scale bar, 50 μm. **g** TUNEL staining in liver sections from AD-shNC and AD-shKLF6 mice after I/R. Scale bar, 20 μm. Each group includes six mice. Mean values ± SD are shown, and statistical significance was determined by two-tailed Student’s *t* test. *p < 0.05.

To further investigate the role of KLF6 in hepatic I/R injury, murine KLF6 was knocked down through adenoviral delivery of shRNA via tail vein injection. The efficiency of KLF6 knockdown was confirmed by RT-qPCR (Fig. 2a) and western blotting analysis (Fig. 2b). Notably, H&E staining revealed a significant increase in necrotic area in liver sections of AD-shKLF6 mice compared to AD-shNC mice following I/R (Fig. 2c). In agreement with the results of histology, KLF6 knockdown significantly enhanced the levels of the common serum markers of liver damage (ALT, AST, and LDH) (Fig. 2d). These results demonstrated that KLF6 deficiency exacerbated I/R-mediated liver insult.

Inflammatory responses play important roles in the initiation and progression of hepatic I/R injury [19]. Since KLF6 has been shown to be involved in inflammatory responses [20], we next examined whether KLF6 affected the inflammatory responses in mice following hepatic I/R injury. As measured by ELISA, the AD-shKLF6 group had significantly higher serum TNF-α, IL-6, and CXCL2 levels when compared to the AD-shNC group (Fig. S2a). These modifications were also confirmed by RT-qPCR in liver tissue (Fig. S2b). Moreover, the infiltration of Ly-6G^+^ neutrophils (Fig. 2e) and F4/80^+^ macrophages (Fig. 2f) into hepatic tissues was significantly enhanced in the AD-shKLF6 group in comparison to the control group. The TUNEL assay evaluation of hepatocyte apoptosis following I/R revealed a greater number of TUNEL-positive cells in AD-shKLF6 mice than in the AD-shNC mice (Fig. 2g). Collectively, the preceding observations indicate that KLF6 deficiency exacerbated liver I/R-induced injury and inflammation.

### KLF6 overexpression ameliorates liver damage and inflammation induced by hepatic I/R insult

**Fig. 3:**
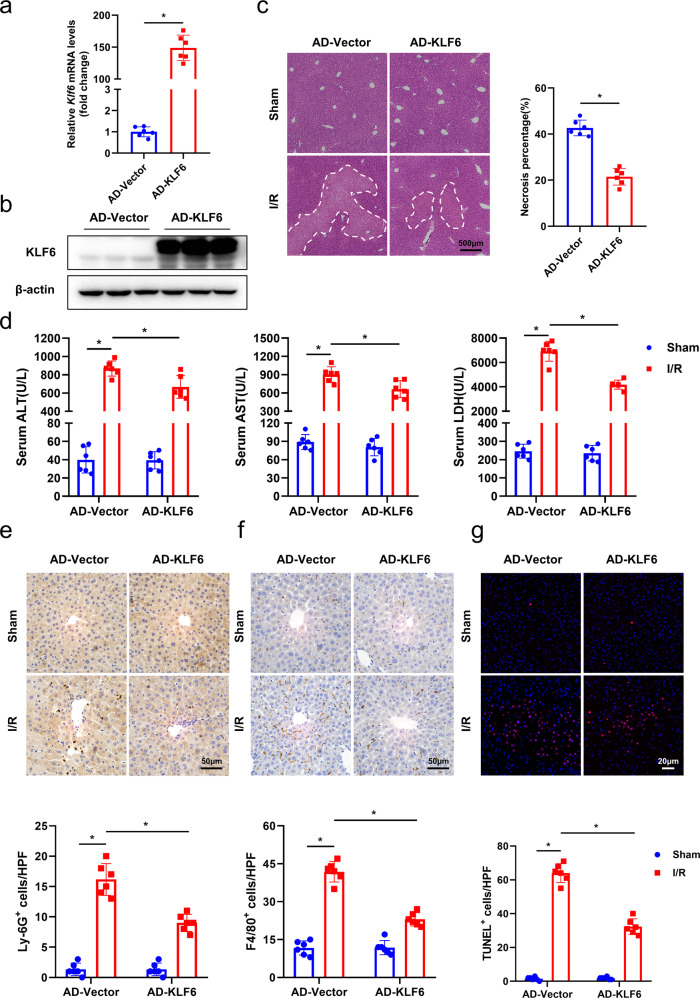
KLF6 overexpression ameliorates hepatocellular injury and inflammation induced by hepatic I/R. **a**, **b** Verification of KLF6 overexpression after tail vein injection of adenoviruses by RT-qPCR and western blotting analysis. **c** Liver sections were stained by H&E staining, with the outlined areas showing hepatic necrosis. Scale bar, 500 μm. **d** Serum ALT/AST/LDH was detected after hepatic I/R surgery. **e**, **f** Neutrophils (Ly6G^+^) and macrophages (F4/80^+^) were identified. Scale bar, 50 μm. **g** Representative images of the TUNEL staining. Scale bar, 20 μm. Each group includes six mice. Mean values ± SD are shown, and statistical significance was determined by two-tailed Student’s *t* test. *p < 0.05.

Given that knocking down KLF6 can aggravate hepatic I/R injury, we were interested in determining whether its overexpression protects the liver from I/R-induced insult. Accordingly, we used a recombinant adenovirus to overexpress KLF6 (AD-KLF6) in mice. Consistent with RT-qPCR findings, the results of the western blotting analysis demonstrated a marked increase in KLF6 protein expression (Fig. 3a, b). As expected, the overexpression of the KLF6 in AD-KLF6 mice resulted in a significant decrease in the I/R-induced necrotic area in comparison to the control mice (AD-Vector) (Fig. 3c). Serum levels of ALT, ASL, and LDH decreased significantly after KLF6 overexpression, indicating decreased liver damage (Fig. 3d). In addition, elevated KLF6 expression weakened I/R-induced inflammation. As a result, the AD-KLF6 mice demonstrated decreased mRNA and protein levels of pro-inflammatory cytokines/chemokines, including TNF-α, IL-6, and CXCL2 (Fig. S3a, b) in comparison to the AD-Vector mice. Furthermore, a decreased infiltration of Ly-6G^+^ neutrophils (Fig. 3e) and F4/80^+^ macrophages (Fig. 3f) in the livers of AD-KLF6 mice was observed after hepatic I/R surgery, when compared to the AD-Vector mice. Accordingly, the TUNEL staining of sections of AD-KLF6 mice revealed fewer positively-stained nuclei compared to the control group (Fig. 3g). Collectively, these findings demonstrated that KLF6 overexpression in liver ameliorated hepatocellular injury and inflammation induced by hepatic I/R treatment.

### KLF6 deficiency aggravates H/R‑induced AML12 cell injury

**Fig. 4:**
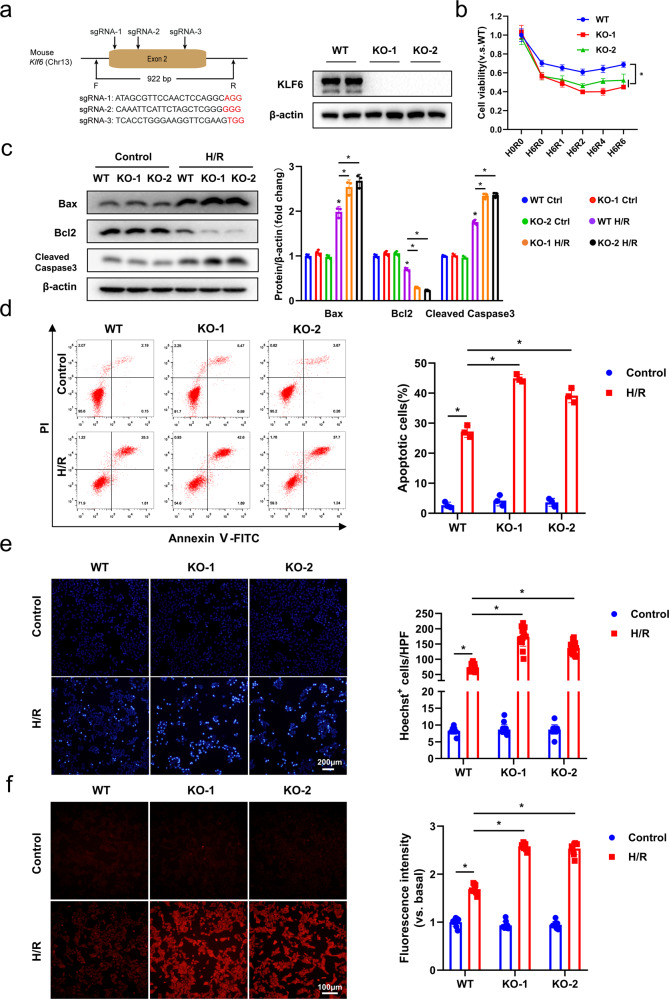
KLF6 deficiency aggravates H/R‑induced AML12 cell injury. **a** Left panel, schematic of CRISPR/Cas9-mediated knockout of *Klf6* in AML12 cells. Right panel, western blotting analysis of KLF6 expression in WT and KO AML12 cells. **b** WT and KO cells were exposed to 1% O_2_ for 6 h, and then they were transferred to normoxia and the cell viability was measured by CCK8 kit at different time points after reoxygenation. **c** Expression of cleaved caspase3, BAX and Bcl2 were detected by western blot analysis. **d** Left panel, representative flow cytometry plots of annexin V/PI stained cells. Right panel, statistical analysis of total apoptotic cells in different groups. **e** Nuclei were visualized with Hoechst 33342. Apoptotic cells were distinguished by the presence of bright blue nuclei. Scale bar, 200 μm. **f** In situ fluorescent staining (red) detection of ROS in KLF6 WT and KO cells after H/R (Left panel) and quantified average fluorescence intensity by ImageJ (Right panel). Scale bar, 100 μm. Mean values ± SD are shown, and statistical significance was determined by one-way ANOVA followed by Dunnett’s multiple comparison test. *p < 0.05.

After demonstrating that KLF6 was localized to hepatocytes and played a crucial role in I/R-induced liver injury, we utilized the AML12 murine liver cell line to further evaluate its biological functions and the underlying molecular mechanism. We generated two KLF6 KO AML12 cell lines using the CRISPR/CAS9 system. As shown in Fig. 4a, the loss of KLF6 protein expression in the two KO cell lines was confirmed by western blotting analysis. WT and KO cells were then subjected to 6 h of hypoxia, followed by reoxygenation for the indicated periods. As demonstrated by the CCK8 assay, KLF6 depletion significantly decreased cell viability compared to WT cells (Fig. 4b). Because the effects of H/R on cell viability were greatest at 6 h of hypoxia and 2 h of reoxygenation, this time frame was selected for subsequent experiments.

To determine whether KLF6 influenced apoptosis induced by H/R, we examined the expression of specific cell death markers using western blotting analysis. We observed a decrease in BCL2 (pro-survival factor) expression and an increase in cleaved caspase-3 and BAX (pro-apoptotic factors) expression in KO cells relative to WT cells (Fig. 4c). In addition, flow cytometry (FCM) was used to examine the effect of KLF6 on cell apoptosis. For each sample, the percentage of total apoptotic cells was quantified as the sum of early apoptotic (annexin V-positive) and late apoptotic cells (annexin V-PI-double positive). Compared to the control groups, the KLF6 KO cells exhibited a significantly elevated apoptosis rate (Fig. 4d). Subsequently, Hoechst 33342 staining was used to evaluate apoptotic cells, and consistent conclusions were inferred (Fig. 4e).

By activating numerous signaling pathways, ROS plays a pivotal role in I/R-induced hepatocyte death [21]. We examined the ROS levels in WT and KO cells subjected to H/R using DHE fluorescent probes. Although the depletion of KLF6 did not affect the basal levels of ROS in normoxia, the increase in ROS levels during H/R was more pronounced in KO cells compared to WT cells (Fig. 4f). This indicated that KLF6 contributed to the ability of cells to respond to oxidative stress. Consistent with the finding in vivo, KLF6 knockout in AML12 cells significantly promoted the expression of pro-inflammatory factors (*Tnf-α*, *Il-6*, and *Cxcl2*) (Fig. S4a).

AML12 cells with a KLF6 deficiency exhibited elevated apoptosis, inflammatory response, and ROS production, as suggested by above findings.

### KLF6 overexpression attenuates H/R‑induced AML12 cell injury

**Fig. 5:**
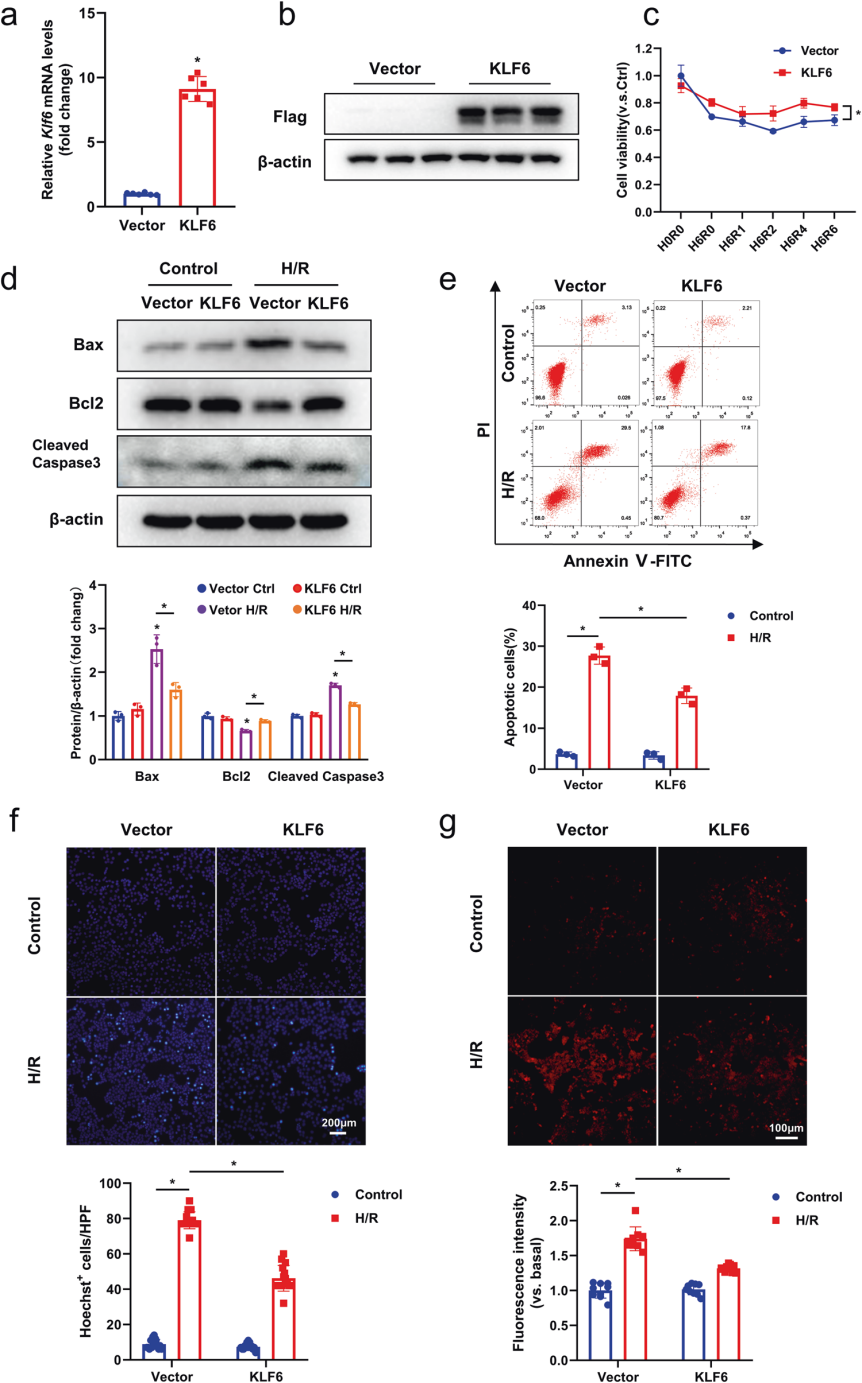
KLF6 overexpression attenuates H/R‑induced AML12 cell injury. **a**, **b** Western blotting and RT-qPCR analysis of KLF6 expression of Vector and KLF6 cells. **c** The viability of Vector and KLF6 cells following hypoxia and reoxygenation was evaluated by a CCK8 assay at the indicated time points. **d** Western blotting analysis for cleaved caspase3, BAX and Bcl2 after H/R in the Vector and KLF6 cells. **e** Upper panel, representative flow cytometry plots of annexin V/PI stained cells. Lower panel, statistical analysis of total apoptotic cells in different groups. **f** Representative pictures (upper panel) and quantitative analysis (lower panel) of nucleus stained with Hoechst 33342. Scale bar, 200 μm. **g** In situ fluorescent staining (red) detection of ROS in Vector and KLF6 cells after H/R (upper panel) and quantified average fluorescence intensity by ImageJ (lower panel). Scale bar, 100 μm. Vector AML12 cells were infected with an empty control lentiviral vector, KLF6 AML12 cells were infected with a lentiviral construct expressing KLF6. Mean values ± SD are shown, and statistical significance was determined by two-tailed Student’s *t* test. *p < 0.05.

After demonstrating the potentially detrimental effects of KLF6 knockout on AML12 cells, we overexpressed KLF6 to further evaluate its significance. KLF6 was, therefore, overexpressed in AML12 cells using a lentivirus system. Correspondingly, we verified the effect of KLF6 overexpression via RT-qPCR (Fig. 5a) and western blotting (Fig. 5b) analyses. The CCK8 assays demonstrated that overexpression of KLF6 significantly increased cell viability in comparison to the control lentivirus group (Fig. 5c). Moreover, the western blotting results revealed elevated levels of Bcl2, along with a significant decrease in the expressions of Bax and cleaved-caspase3 in the KLF6 overexpression group, when compared to the Vector group (Fig. 5d). Subsequently, the effects of KLF6 on the apoptosis of AML12 cells were determined by flow cytometry (Fig. 5e). After KLF6 overexpression, the number of apoptotic cells significantly decreased, confirming our hypothesis. Quantitative analysis of Hoechst 33342 nuclear staining yielded similar results (Fig. 5f). Using the ROS-sensitive fluorescent dye dihydroethidium (DHE) to detect intracellular ROS levels, we found that the increased expression of KLF6 decreased intracellular ROS levels after H/R (Fig. 5g). Accordingly, the increased transcript levels of inflammatory factors (*Tnf-α*, *Il-6*, and *Cxcl2*) in response to H/R were reversed by the overexpression of KLF6 (Fig. S4b).

In summary, these in vivo and in vitro studies demonstrated that KLF6 in hepatocytes regulated the damage response to hepatic I/R.

### Autophagy inhibition is critically involved in the protective effect of KLF6 on AML12 cells against H/R injury

The data presented above demonstrated both in vitro and in vivo protective effects of KLF6 during I/R. However, the underlying molecular mechanism remained unclear. As previously reported, the KLF family is involved in autophagy regulation [17, 18]. Autophagy also plays a crucial role in liver I/R injury [15]. Therefore, we hypothesized that KLF6 may play an important role in liver I/R injury by regulating autophagy.

**Fig. 6:**
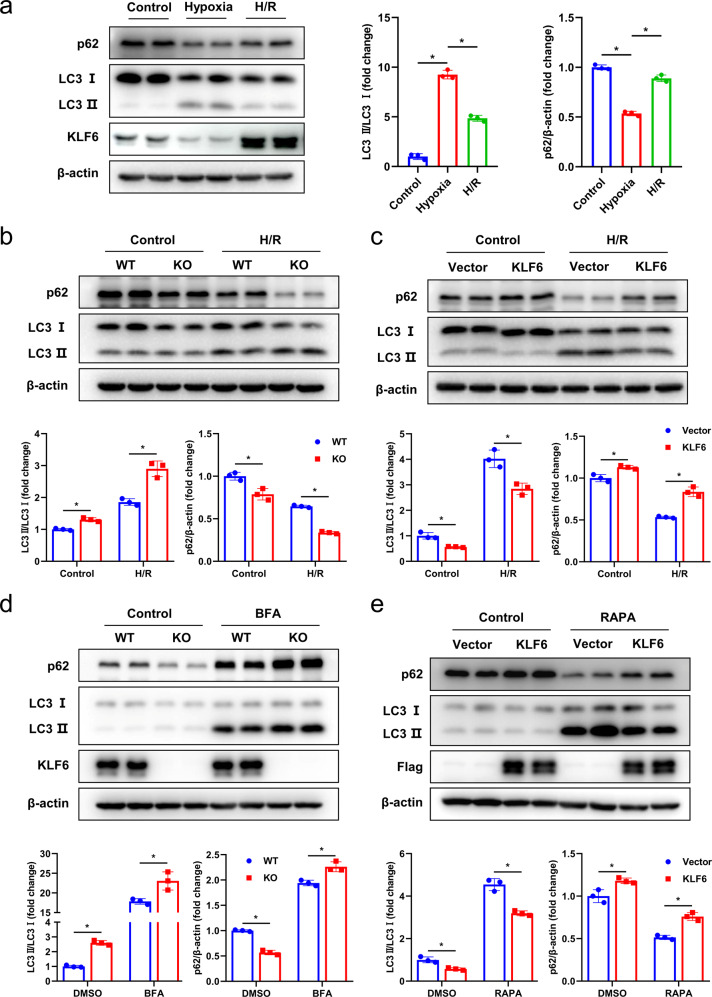
KLF6 inhibits autophagy initiation in AML12 cells. **a** Western blotting analysis for LC3, p62 and KLF6 levels in AML12 cells exposed to normoxic control (Control), 6 h hypoxia (Hypoxia), 6 h hypoxia followed by 2 h reoxygenation (H/R). **b** Western blotting analysis for LC3, p62 in wild-type AML12 cells and KLF6 knockout cells exposed to normoxic control (Control) or 6 h hypoxia followed by 2 h reoxygenation (H/R). **c** Western blotting analysis for LC3, p62 in KLF6-vector lentivirus cells and KLF6-overexpression lentivirus cells subjected to normoxic control or H/R. **d** WT and KO cells were pre-treated with 400 nmol/l BFA for 12 h. Western blotting was performed with indicated antibodies. **e** KLF6-vector lentivirus cells and KLF6-overexpression lentivirus cells were pre-treated with 10 μmol/l RAPA for 24 h. Western blotting was performed with indicated antibodies. Mean values ± SD are shown, and statistical significance was determined by two-tailed Student’s *t* test. *p < 0.05.

**Fig. 7:**
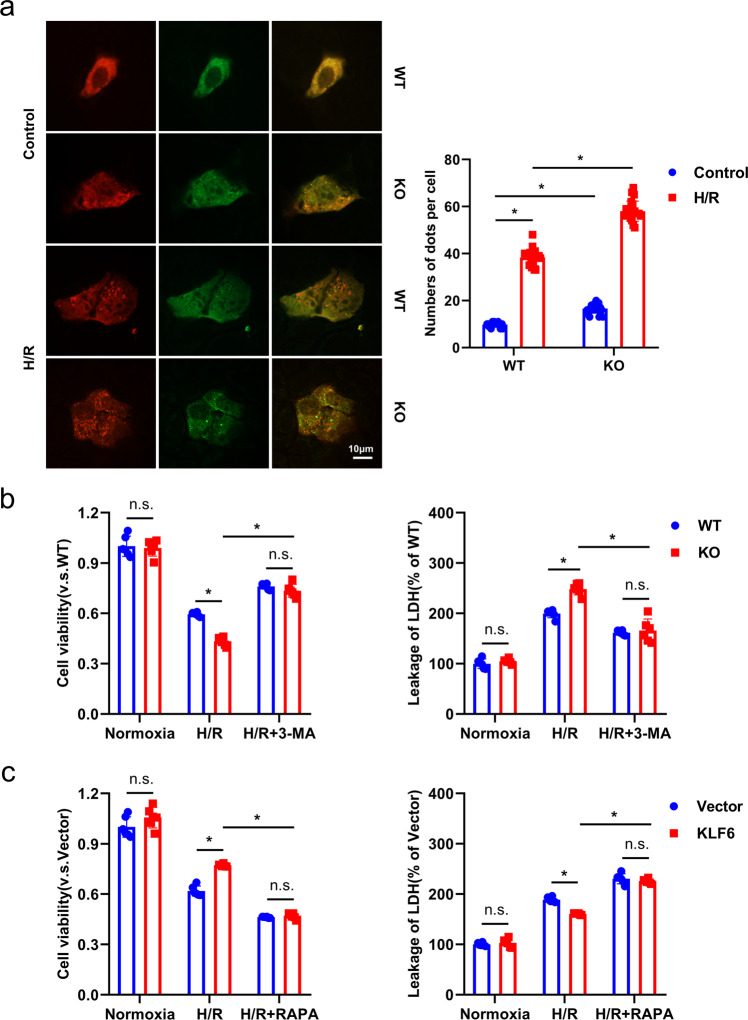
The regulatory effects of KLF6 on H/R injury in AML12 cells are autophagy dependent. **a** The autophagic flux of mCherry-GFP-LC3 transfected WT and KO cells after normoxic control or H/R was revealed using laser confocal microscopy (left panel) and assessed by visual screening (right panel). Scale bar, 10 μm. **b**, **c** Cells were pre-treated with 3-MA (5 mmol/l, 1 h) or RAPA (10 μmol/l, 24 h) and then subjected to H/R. Cell viability and LDH release were evaluated with the CCK8 Assay Kit and LDH Assay Kit, respectively. Mean values ± SD are shown, and statistical significance was determined by two-tailed Student’s *t* test. *p < 0.05.

Initially, we detected the expression of KLF6 and the autophagy-related proteins LC3 and SQSTM1/p62 during H/R by western blotting analysis. Increasing LC3-II levels and decreasing SQSTM1/p62 levels were predicted if autophagy was indeed activated [22]. As depicted in Fig. 6a, the level of autophagy increased significantly following hypoxia and partially recovered following reoxygenation. However, KLF6 displayed a strikingly opposite trend. We then investigated whether KLF6 could influence autophagy flux. In both normoxia and H/R models, the autophagy level of AML12 cells increased significantly after KLF6 knockout (Fig. 6b). Furthermore, overexpression of KLF6 could significantly reduce the level of autophagy in normoxia and after H/R stress (Fig. 6c). In addition, we pre-treated the cells with an autophagy inhibitor, bafilomycin A1 (BFA), or an autophagy activator, rapamycin (RAPA). LC3-I lipidation and SQSTM1/p62 increased significantly upon BFA treatment in KLF6-deficient cells (Fig. 6d); conversely, the overexpression of KLF6 inhibited RAPA-induced autophagy activation (Fig. 6e). These findings indicate that KLF6 is capable of inhibiting the activation of autophagy at the initial stage. We confirmed this observation using a tandem mCherry-GFP-LC3 fluorescence analysis; autophagosomes appear as yellow (mCherry and GFP) puncta, whereas autolysosomes appear as red (mCherry) puncta. We observed that KLF6 KO cells contained a greater number of puncta, predominantly red, indicating autophagy induction. After H/R, this finding became more prominent (Fig. 7a). Thus, our results demonstrated that KLF6 was a negative regulator of autophagy.

To further clarify whether KLF6 exerted its protective effect on AML12 cells by regulating autophagy, KLF6 knockout cells were treated with the autophagy initiation inhibitor, 3-methyladenine (3-MA). We observed that 3-MA treatment prevented the cytotoxic effects of KLF6 knockout on AML12 cells exposed to H/R, as evidenced by an increase in cell viability and a reduction in LDH release (Fig. 7b). In addition, the presence of RAPA completely reversed the reduction in H/R-induced cell death and LDH release from KLF6-overexpressing cells (Fig. 7c). Collectively, these findings indicate that the protective effects of KLF6 against H/R-induced injury to AML12 cells were mediated by the inhibition of autophagy.

### KLF6 regulates autophagy through the direct inhibition of Beclin1 gene transcription and activation of the mTOR/ULK1 signaling pathway

**Fig. 8:**
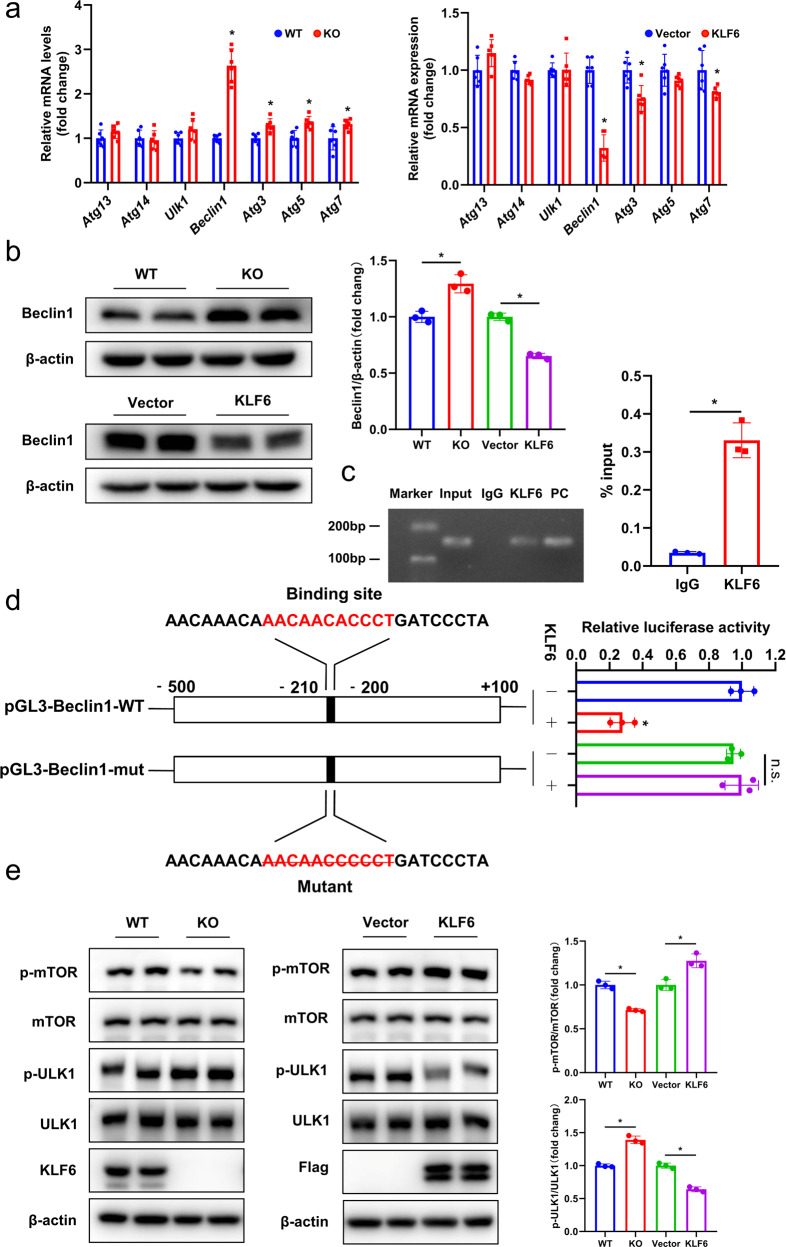
KLF6 suppresses the overactivation of autophagy by transcriptional regulation of Beclin1 and activation of the mTOR/ULK1 pathway. **a** After KLF6 was knocked out and overexpressed, the gene expression of key molecules of autophagy pathway *Atg13*, *Atg14*, *Ulk1*, *Beclin1*, *Atg3*, *Atg5*, *Atg7* was detected by RT-qPCR. **b** Western blotting analysis of Beclin1 after KLF6 knockout or overexpression. **c** ChIP-qPCR showed the binding of KLF6 to the promoter regions of *Beclin1*. The DNA ladder (Marker), genomic DNA (Input), IgG ChIP (IgG), KLF6 ChIP (KLF6), and the positive control group (PC) are also presented at right. **d** Schematic diagrams showed the mutation sites of luciferase reporter gene vector (left panel). AML12 cells were co-transfected with pGL3-Beclin1-WT or pGL3-Beclin1-mut luciferase vector and KLF6 expression vector or control vector, and then dual luciferase assay was performed. Quantification is presented in the right panel. **e** Western blotting analysis of KLF6, ULK1, p-ULK1, mTOR, p-mTOR in each group. WT wild-type AML12 cells, KO KLF6 knockout AML12 cells, Vector AML12 cells were infected with an empty control lentiviral vector, KLF6 AML12 cells were infected with KLF6 overexpression lentiviruses. Mean values ± SD are shown, and statistical significance was determined by two-tailed Student’s *t* test. *p < 0.05.

To determine the mechanism by which KLF6 inhibits autophagy in AML12 cells, we examined the effect of KLF6 on the expression of key autophagy-related genes, including *Atg5*, *Atg7*, *Beclin1*, and *Ulk1*. Among these tested genes, *Beclin1* showed the most significant change when KLF6 was knocked out or overexpressed in AML12 cells (Fig. 8a). Moreover, KLF6 and Beclin1 expressions exhibited opposing trends in relation to one another (Fig. 8a, b), which was consistent with our earlier findings that demonstrated the negative regulation of autophagy by KLF6. To further validate our findings, we overexpressed KLF6 in KO cells using a lentiviral vector system and observed that the KLF6 KO-induced increase in Beclin1 level was dramatically reversed (Fig. S5a). Our in vivo data are in concordance with our in vitro results, suggesting that Beclin1 was significantly increased after hepatic I/R and KLF6 could inhibit Beclin1 expression (Fig. S5b, c). Given that KLF6 is a transcription factor, we investigated whether its regulation of Beclin1 occurred at the transcriptional level. As demonstrated in Fig. 8c, ChIP-qPCR confirmed the enrichment of KLF6 on the *Beclin1* gene promoter. Through sequence alignment on the JASPAR website (http://jaspar.genereg.net/analysis), a potential KLF6 binding site (red) was predicted with strong confidence in the promoter of *Beclin1* (score 0.91). Using the wild-type pGL3-Beclin1 plasmid as a template, site-directed mutations were then generated to determine the function of the predicted site. Luciferase reporter assay showed that KLF6 overexpression significantly decreased luciferase activity in AML12 cells transfected with pGL3-Beclin1-WT but not pGL3-Beclin1-mut (Fig. 8d).

The mTOR signaling pathway plays a central role in the regulation of autophagy [23]. To further investigate the mechanism of KLF6’s regulation of autophagy, a western blotting analysis was performed to determine the impact of KLF6 knockout or overexpression on the mTOR/ULK1 signaling pathway. Compared to the WT group, there was no significant difference in the total protein levels of mTOR and ULK1 following KLF6 knockout; however, the levels of phosphorylated m-TOR(p-mTOR) decreased markedly, while that of p-ULK1 increased significantly. In response to the overexpression of KLF6, the total protein content of mTOR and ULK1 did not differ significantly from the control group, but the expression of p-mTOR increased significantly, whereas that of p-ULK1 decreased significantly (Fig. 8e). These findings indicate that KLF6 may activate the mTOR/ULK1 signaling pathway.

### The expression of KLF6 correlates significantly with liver function after clinical LT

**Fig. 9:**
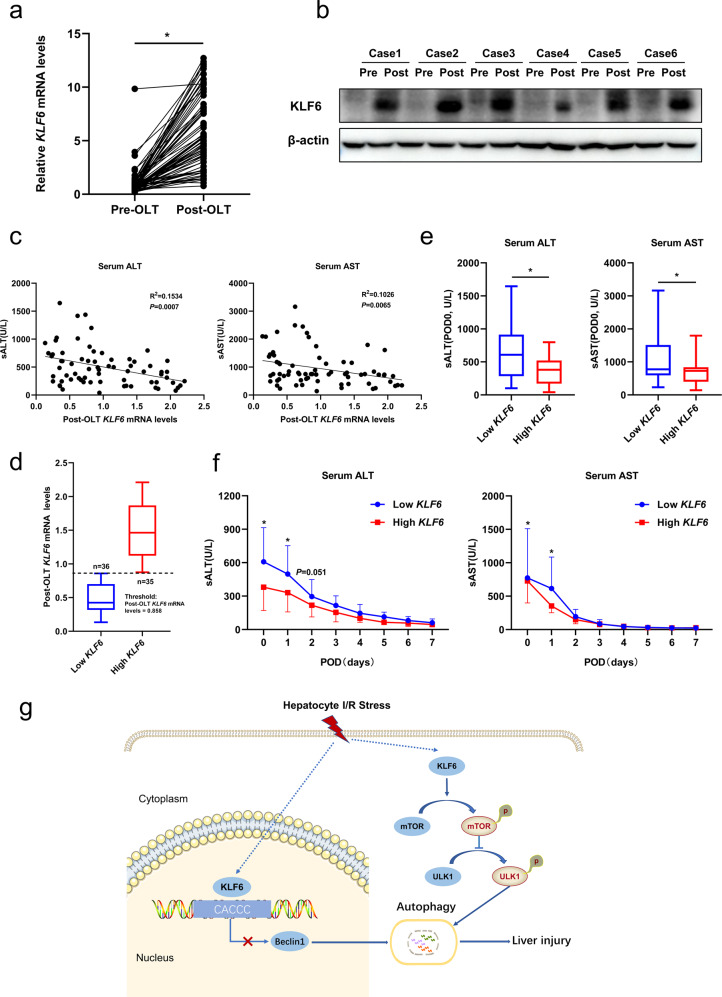
The expression of KLF6 associates significantly with liver function after clinical liver transplantation. **a** RT-qPCR detection of *KLF6* gene expression in liver tissues pre- and post-orthotopic liver transplant (OLT). **b** Western blotting analysis of KLF6 in liver tissues pre- and post-OLT. **c** Correlation analysis between post-OLT *KLF6* mRNA levels and postoperative serum ALT and AST (POD0). **d** Patients were divided into high and low expression group according to the median of post-OLT *KLF6* mRNA levels. **e** Comparison of serum ALT and AST levels (POD0) between *KLF6* low expression group and *KLF6* high expression group. **f** The line chart of the change of serum ALT and AST with postoperative days (POD) in the *KLF6* low expression group and *KLF6* high expression group. **g** Schematic illustration of the mechanism of KLF6 regulating liver ischemia-reperfusion injury. Mean with interquartile range are shown, and statistical significance was determined by Wilcoxon matched-pairs signed rank test (**a**), two-sided non-parametric Spearman rank correlation tests (**c**) or Mann–Whitney *U* tests (**e**, **f**). *p < 0.05.

We detected the expression of KLF6 in liver tissues prior to and following transplantation using RT-qPCR and western blotting analysis. As shown in Fig. 9a, b, the gene and protein expressions of KLF6 following transplantation were significantly higher than those prior to LT. The correlation between the mRNA level of *KLF6* in liver tissues after liver transplantation and the postoperative liver function of the patients was further analyzed and revealed a significant negative correlation with serum ALT and AST (POD0) (Fig. 9c). Correspondingly, we divided the patients into two groups based on the median value of post-orthotopic liver transplantation (OLT) *KLF6* mRNA levels: *KLF6* low expression (*n* = 36) and *KLF6* high expression (*n* = 35) groups (Fig. 9d). Table S2 displays the comparability of the baseline characteristics of the two patient groups. After LT, the serum levels of ALT and AST in the *KLF6* high expression group were significantly lower than in the *KLF6* low expression group (Fig. 9e). Short-term liver function in the *KLF6* high expression group was significantly superior to that of the *KLF6* low expression group, as depicted by the line chart of liver function changes with time after LT (Fig. 9f).

## Discussion

As a result of advances in surgical technology and the use of immunosuppressants, LT has emerged as the most effective treatment for almost all types of end-stage liver disease. I/R injury is a major prognostic factor for LT patients [24]. In this study, KLF6 expression was significantly increased in vivo, in vitro, and in clinical specimens after hepatic ischemia-reperfusion, suggesting its role as a key regulator of hepatic I/R injury. KLF6 expression is upregulated after renal I/R injury, but the underlying molecular mechanism remains unclear [25]. Through gain- and loss-of-function experiments for KLF6 expression, we determined that it plays an essential role in the regulation of liver I/R injury. Importantly, our results indicated that KLF6 inhibited the overactivation of autophagy via transcriptional regulation of Beclin1 and activation of the mTOR/ULK1 pathway, thereby protecting the liver from I/R injury (Fig. 9g). KLF6 may thus be a promising molecule for protecting the liver against I/R-mediated hepatocellular damage in transplant recipients.

Currently, hepatocyte apoptosis, necrosis, inflammatory responses, and ROS levels are used to assess the severity of liver ischemia-reperfusion injury. KLF6 plays an important regulatory role in cell survival and apoptosis. Silencing KLF6 with siRNA can result in cell cycle arrest and increase DNA damage-induced apoptosis [26]. Sirach et al. reported that KLF6 inhibits the apoptosis of liver cancer cells [27]. Horne et al. discovered that podocyte-specific deletion of KLF6 increased mitochondrial damage and apoptosis [13]. In accordance with these reports, our in vitro an in vivo experiments demonstrated that KLF6 could inhibit hepatocyte apoptosis and necrosis after liver I/R injury.

The regulatory role of KLF6 in inflammatory responses is still debatable. Kim et al. [28] reported that KLF6 promoted inflammatory and hypoxic responses in macrophages. In contrast, KLF6 was able to transactivate multiple genes that negatively regulated the NF-κB pathway, thereby decreasing the nuclear localization of NF-κB and downregulating the expression of its target genes in glioblastoma [29]. We discovered that KLF6 inhibited the mRNA and protein expression of inflammatory factors such as TNF-α, IL-6, and CXCL2, as well as the infiltration of neutrophils and macrophages in liver tissues caused by I/R injury. Consistent with our findings, inhibiting KLF6 expression in keratinocytes increased inflammatory responses during aging and accelerated cellular senescence [30]. Azilsartan attenuates oscillatory shear stress-induced endothelial dysfunction and inflammatory responses by upregulating KLF6 [31]. Consequently, different cell types, disease models, or stimulation conditions may play a role in the dual regulation of KLF6 on inflammatory responses.

Herein, KLF6 knockout significantly increased, while its overexpression markedly reduced ROS levels in AML12 cells after hypoxia/reoxygenation. Recent studies have also demonstrated that febuxostat inhibits oxidative stress and inflammatory responses by upregulating KLF6, thereby reducing the toxicity of propofol to brain endothelial cells [32]. Sitagliptin reduces high glucose-induced oxidative stress and inflammation and increases the permeability of renal tubular endothelial cells by promoting KLF6 expression [33]. These studies indicate that KLF6 can reduce the level of cellular oxidative stress and enhance their ability to respond to damaging stimuli.

Although autophagy, apoptosis, and inflammatory responses are distinct pathophysiological processes, they can occur simultaneously in liver I/R injury, influencing one another, and jointly regulating the onset and progression of diseases. Autophagy-related genes activate the Fas-dependent death-inducing signaling complex, thereby promoting the expression of apoptotic genes and initiating the process of apoptosis [34]. By activating the PI3K/Akt signaling pathway, ATG3 knockout inhibits autophagy and attenuates glucose-oxygen deprivation-induced damage and inflammation in brain microvascular endothelial cells [35]. Overexpression of IL37, an anti-inflammatory factor, inhibits hepatic I/R injury-induced autophagy and apoptosis by regulating the AMPK/mTOR/ULK1 axis [36]. Given the importance of the KLF family proteins in autophagy regulation, we hypothesized that KLF6 protected the liver from I/R injury by regulating autophagy.

First, we found that KLF6 and autophagy exhibited opposite trends during H/R in AML12 cells. Furthermore, by knocking out and overexpressing KLF6, using autophagy agonists and inhibitors, and transfecting Ad-mCherry-GFP-LC3B recombinant adenovirus to detect autophagic flux, it was demonstrated KLF6 inhibits autophagy activation at the initial stage. Following 3-MA preconditioning, the hepatocyte-damaging of KLF6 knockout was reversed. Moreover, after pretreatment with RAPA, overexpression of KLF6 showed no significant protective effect on hepatocytes against H/R. These experiments revealed that KLF6 protected AML12 cells from H/R injury by inhibiting the overactivation of autophagy. In this regard, the diverse roles of the KLF family in regulating autophagy have been reported previously. Hsieh et al. [17] demonstrated that KLF4 improved vascular endothelial cell autophagy and vascular function in aged mice. Moreover, in a mouse arthritis model, the level of KLF2 in monocytes was reduced, which in turn promoted their differentiation into osteoclasts by activating autophagy [37]. In addition, knocking down KLF5 was reported to desensitize castration-resistant prostate cancer cells to docetaxel by inducing autophagy [38].

Beclin1, a central component of the class III phosphatidylinositol 3-kinase (PI3K-III) complex, is essential for membrane trafficking and reorganization of autophagy [39]. During the reperfusion phase following myocardial ischemia, the expression of the Beclin1 protein and autophagy levels are significantly upregulated [40]. After inhibiting the expression of Beclin1 with siRNA, the level of autophagy and the degree of apoptosis in cardiomyocytes after I/R injury decreased substantially [41]. However, the mechanism underlying the upregulation of Beclin1 in hepatic I/R injury remains unknown. Using the promoter luciferase assay and ChIP-qPCR, we first demonstrate that KLF6 could inhibit the expression of *Beclin1* by binding to the “CACCC” motif in the promoter region. KLF2 repressed Beclin1 transcription by decreasing histone H4K8 and H3K9 acetylation levels [37], which is consistent with our findings. In prostate cancer cells, KLF5 collaborates with HDAC3 to inhibit *Beclin1* transcription and reduce autophagy [38]. Interestingly, in HepG2 cells, KLF6 can transcriptionally activate ATG7 and Beclin1 in a p53-dependent manner, thereby increasing the level of autophagy [42], which contradicts our findings. We hypothesized that in various cell lines and experimental models, KLF6 might play a bidirectional regulatory role in autophagy, either alone or in concert with other regulators.

The mTOR signaling pathway is a central regulator of autophagy and regulates multiple steps of the autophagy process, including nucleation, autophagosome elongation, autophagosome maturation, and termination [43]. mTORC1 is capable of phosphorylating ULK1 at the P757 site, interfering with the interaction between AMPK and ULK1, and inhibiting autophagy initiation [44]. Through the transcriptional regulation of PDGFB, Syafruddin et al. [12] discovered that KLF6 activated the mTOR signaling pathway and affected lipid metabolism, thereby promoting the progression of renal clear cell carcinoma. In this study, KLF6 was able to activate the mTOR/ULK1 signaling pathway without affecting the total protein expression of mTOR and ULK1, thereby regulating the autophagy level in AML12 cells.

The discovery of a sensitive biomarker to evaluate the severity of I/R injury has significant clinical implications. Huang et al. conducted combined transcriptome and proteome analyses in LT patients using bioinformatics, suggesting that KLF6 may be one of the key regulators involved in liver I/R injury; however, this has not yet been experimentally confirmed [45]. In the current study, KLF6 was found to be significantly upregulated after LT for the first time. In addition, the expression of *KLF6* was negatively correlated with serum liver enzymes of patients after LT, and the liver function of patients with high *KLF6* expression was significantly superior to than that of patients with low *KLF6* expression. It is hypothesized that KLF6 could serve as a biomarker for predicting the severity of liver I/R injury following LT.

In addition to KLF6, other KLF proteins also play important roles in different liver diseases. KLF9 promotes PGC1α expression and activates the hepatic gluconeogenic program, which is an underlying pathogenesis of glucocorticoid therapy-induced diabetes [46]. In non-alcoholic fatty liver disease (NAFLD), KLF16 improves steatohepatitis and insulin resistance by targeting PPARα [47]. Although KLF proteins can be divided into three different functional subgroups [48], whether there is synergy or antagonism between different KLF proteins needs further study.

Overall, our findings identify KLF6 as a novel protective regulator in the liver subjected to I/R surgery. Moreover, KLF6 inhibits the overactivation of autophagy via transcriptional regulation of Beclin1 and activation of the mTOR/ULK1 pathway, thereby protecting AML12 cells from H/R injury. Our findings, therefore, provide crucial insights into developing a novel therapeutic target and a useful biomarker for hepatic I/R injury.

## Materials and methods

### Clinical liver transplantation study

We performed a retrospective observational cohort study who underwent OLT at the First Affiliated Hospital of Zhengzhou University, from September 2018 through to November 2019. After excluding deaths and cases with incomplete clinical data, a total of 71 cases were included in the study. All grafts were retrieved from brain*-*death donors (DBD) and signed informed consent. According to standard operating procedures, the donor liver was evaluated, trimmed, an orthotopic liver transplantation was performed, and immunosuppressive therapy was administered postoperatively. Non-fatty donor organs were stored in UW solution prior to OLT. The duration between the donor’s perfusion and the end of cold preservation was defined as the cold ischemia time. To evaluate liver function, serum ALT (sALT) and AST (sAST) were obtained from the inpatient register system. A total of nine time points were included: prior to transplantation, from postoperative day 0 (POD0, collected at the time of ICU arrival) to postoperative day 7 (POD7). The pre-transplant liver specimens were obtained from the left lobe of the liver when trimming the donor liver. The post-transplant liver specimens were acquired prior to the abdominal closure. Subsequently, the specimens before and after transplantation were analyzed by Western blotting and RT-qPCR. Our study was approved by the Ethics Committee of the First Affiliated Hospital of Zhengzhou University and all patients signed informed consent forms (ethics number:2018-KY-73).

### Mouse warm liver I/R injury model

Male C57BL6/N mice (6–8 weeks old) were purchased from VitalRiver (Beijing, China). Mouse partial (70%) warm ischemia reperfusion (I/R) injury model was performed as previously described [49]. In general, an atraumatic microvascular clip was utilized to interrupt the blood supply of the left and middle liver lobes (70%) for 60 min. Reperfusion was achieved by releasing the vascular clip, and mice were sacrificed 6 h later. Liver tissues and serum were collected for subsequent analysis. Mice were randomly divided into six per group. In order to overexpress or inhibit hepatic KLF6 expression, adenoviruses (1 × 10^9^ pfu per mouse in 200 μl saline) were injected through the tail vein. The I/R model was established 7 days after the adenovirus injection.

### Construction of adenoviral vectors and lentiviral vectors

The overexpressing and suppressing adenovirus and their respective negative controls were designed, validated, and synthesized by Hanbio (Shanghai, China). The selected interference vector was pAdEasy-U6-CMV-EGFP (EcoRI to MCS), and the pAdEasy-EF1-MCS-3flag-CMV-EGFP vector was used for KLF6 overexpression. Mouse KLF6 shRNA was constructed with top strand of 5′-TCGAGGATCTGAGTTCCTCCGTCATTCTCGAGAATGACGGAGGAACTCAGATCTTTTTTA-3′ and bottom strand of 5′- AGCTTAAAAAAGATCTGAGTTCCTCCGTCATTCTCGAGAATGACGGAGGAACTCAGATCC-3′. Primers used for adenoviral KLF6 overexpression were as follows: forward primer 5′-TGTGACCGGCGCCTACTCTGGTACCGCCACCATGAAACTTTCACCT-3′; reverse primer 5′- CATCGTCATCCTTGTAGTCCTCGAGGAGGTGCCTCTTCATGTGCAG-3′. The KLF6 lentiviral plasmids were constructed by Hanbio (Shanghai, China) using the pHBLV-CMV-MCS-3flag-GSG-P2A-ZsGreen-T2A-PURO vector (forward primer: 5′-TACTAGAGGATCTATTTCCGGTGAATTCGCCACCATGAAACTTTCACCT-3′; reverse primer: AGTCACTTAAGCTTGGTACCGAGGATCCGAGGTGCCTCTTCATGTGCAG-3′).

### Mouse liver function measurement

ALT/AST/LDH levels in mouse serum were measured using a fully automatic biochemical analyzer (Chemray 240; Rayto, Shenzhen, China) in accordance with the manufacturer’s instructions to assess the severity of the liver injury.

### Enzyme-linked immunosorbent assays (ELISA)

The tumor necrosis factor a (TNF-α) ELISA kit (EK282, Liankebio, Hangzhou, China), interleukin 6 (IL-6) ELISA kit (EK206, Liankebio, Hangzhou, China) and C-X-C motif chemokine 2 (CXCL2) ELISA kit (ab204517, Abcam, Cambridge, USA) were used for measuring the levels of mouse serum TNF-α, IL-6 and CXCL2 according to the instructions of the manufacturer.

### Histopathology

Liver tissues were fixed with formalin and then embedded in paraffin and sectioned into a thickness of 4 μm. Hematoxylin and eosin (HE) staining was performed in order to determine the extent of liver necrosis. Terminal deoxynucleotidyl transferase dUTP nick end labeling (TUNEL) assay was performed on tissue sections according to the manufacturer’s instructions (In situ cell death detection kit, TMR red, Roche, Basel, Switzerland). To examine macrophages and neutrophils infiltration, paraffin-embedded sections were immunohistochemically stained with Ly-6G (Servicebio, GB11229) and F4/80 (Servicebio, GB11027) antibodies.

### Cell culture and treatment

The AML12 cell line was acquired from the Cell Bank of the Chinese Academy of Sciences (Shanghai, China). The cells were cultured in DMEM/F12 medium (Gibco, 11330-032) supplemented with 10% fetal bovine serum (Gibco), ITS Liquid Media Supplement (Sigma, I3146), and 40 ng/ml Dexamethasone (Sigma, D4902-100 mg). The lentivirus system was used to generate KLF6-stable overexpression cell lines. The KLF6-KO AML12 cell lines were produced using the CRISPR-Cas9 system described by Luo et al. [50]. To induce anoxia, the media were replaced with serum-free and glucose-free media, and then the cells were placed in an anoxic chamber with 1% oxygen, 5% CO_2_, and balanced N_2_ at 37 °C for 6 h, followed by various reoxygenation periods. As controls, cells were routinely cultured in 21% O_2_ and 5% CO_2_ (normoxic conditions).

### Cell viability

Using the Cell Counting Kit, the influence of H/R on cell viability was determined at the indicated time points (CCK8, Dojindo, Kumamoto, Japan). The cells were plated at 5000 cells/well into 96-well plates. After H/R treatment, 5 µl of CCK8 reagent was added to each well, followed by incubation for 60 min in a humidified incubator. The optical density (OD) value at the wavelength of 450 nm was then measured using a microplate reader (Varioskan LUX, Thermo Fisher, Massachusetts, USA).

### ROS assay and Hoechst 33342 staining

Reactive Oxygen Species Assay Kit (C1300, Applygen, Beijing, China) was used in accordance with the manufacturer’s instructions to measure intracellular ROS levels. Fluorescence was detected via fluorescence microscopy (Olympus IX71, Olympus, Tokyo, Japan). Using an excitation wavelength of 535 nm and an emission wavelength of 610 nm. The positive signals of apoptotic cells were visualized by staining with Hoechst 33342 (C1028, Beyotime, Shanghai, China). Fluorescence was detected at 350 nm excitation wavelength and 461 nm emission wavelength.

### Flow cytometry

Using YF488-Annexin V apoptosis detection kit, apoptosis was measured and quantified by flow cytometry (Y6002, US Everbright Inc., Suzhou, China). Cells (5 × 10^5^ cells/well) were seeded in six-well plates. Following treatment, the cells were pancreatin-digested without EDTA, centrifuged at 300 × *g* for 5 min, and washed twice with pre-cold PBS. After that, the cells were stained with 5 μl annexin V-FITC and 5 μl PI at room temperature and in the dark for 15 min. The stained cells were analyzed on BD FACS Canto II flow cytometer (BD Biosciences, CA, USA).

### Autophagy flux analysis

AML12 WT and KO Cells were plated on glass slides in 24-well plates. After an overnight attachment, the culture was incubated for 24 h with Ad-mCherry-GFP-LC3B adenovirus (C3011, Beyotime Biotechnology, Shanghai, China). Following the indicated treatments, cells were observed using a confocal microscope (Ultraview VOX, Perkin Elmer, Waltham, MA, USA). The yellow puncta (GFP^+^mCherry^+^) represented autophagosomes, whereas the red puncta (GFP^−^mCherry^+^) represented autolysosomes. More than 20 images were randomly selected from three independent experiments and analyzed by ImageJ for quantitative comparisons.

### RNA-seq analysis

TRIzol Reagent was used to extract total RNAs for RNA-seq analysis. Stranded RNA sequencing library preparation was constructed using KCTM Stranded mRNA Library Prep Kit for Illumina® (Catalog No. DR08402, Wuhan Seqhealth Co., Ltd. China) following the manufacturer’s instruction. PCR products corresponding to 200–500 bps were enriched, quantified and finally sequenced (PE150) with Illumina NovaSeq 6000. Clean data were mapped to the mouse reference genome GRCm38 using STRA software (version 2.5.3a) with default parameters. A false discovery rate (FDR)-adjusted *p* value threshold of 0.05 and a fold-change threshold of 2 were employed to identify the set of genes that were significantly upregulated or downregulated. The raw RNA-seq data have been deposited in the National Center for Biotechnology Information (NCBI) with accession code GSE216522.

### Western blot analysis

Western blot experiments were performed as previously described [51]. In brief, whole cell or tissue extracts were prepared with RIPA lysis buffer (Beyotime, P0013B) supplemented with phosphatase inhibitors and protease inhibitors. After lysate incubation for 30 min on ice, supernatant protein was quantified using a BCA kit (Solarbio, PC0020). Equal amounts of protein were subjected to SDS-PAGE and then transferred to nitrocellulose membranes (Millipore, IPFL00010). Membranes were blocked at room temperature for 2 h in 5% milk in TBST, followed by incubation with primary antibodies in 4 °C overnight. Signals were visualized by the imaging system after incubation with secondary antibody.

Antibodies to SQSTM1/P62(#5114), LC3A/B (#12741), Beclin-1(#3495), Phospho-ULK1(Ser757) (#6888), ULK1 (#8054), Phospho-mTOR (Ser2448) (#5536), mTOR (#2983), Bax(#14796), BCL2(#3498), Cleaved Caspase-3(#9664), GAPDH(#2118) were purchased from Cell Signaling Technology (Beverly, MA, USA). Anti-β-actin (#66009-1-Ig) and anti-tubulin (#66031-1-Ig) were purchased from ProteinTech (Wuhan, China). Anti-KLF6 (#sc-365633) was obtained from SantaCruz (Dallas, TX, USA).

### RT-qPCR

Total RNA was extracted using TRIZOL (Invitrogen,15596018). Following RNA quantification and integrity confirmation, total RNA was reversed transcribed into cDNA using HiScript III RT SuperMix(Vazyme, R323-01). qPCR was carried out utilizing the SYBR Green PCR master mix (Vazyme, Q711-02). Primer sequences used in the experiments are listed in Table S1. The level of mRNA expression was normalized with β-actin expression. Each cycle of the qPCR consists of 95 °C (30 s) for denaturing, followed by 40 cycles of 95 °C (10 s) and 60 °C (30 s).

### Luciferase assays

KLF6 WT and KO AML12 cells were seeded at ~45% confluence in 24-well plates the day prior to transfection with luciferase reporter plasmids utilizing lipo3000. Briefly, cells were incubated for 6 h in 250 μl of serum-free medium containing pRL-TK-Renilla (10 ng), pGL3-Beclin1 (200 ng) or pGL3-Beclin1-mut (200 ng). Luciferase activities were measured 24 h later using the Dual-Luciferase Reporter Assay System (#E1910, Promega, Madison, WI, USA) and then normalized to Renilla luciferase values. Measurements were performed three times and results averaged.

### ChIP-qPCR

ChIP followed by quantitative real-time PCR (ChIP-qPCR) was conducted using Simple ChIP Kit (CST, # 9003) following the manufacturer’s standard instructions. In brief, cells were cross-linked in 1% formaldehyde-containing cell culture medium at room temperature for 10 min and quenched with glycine. Chromatin was sheared to ∼200-bp fragments by Micrococcal Nuclease. KLF6 (Santa Cruz, sc-365633) antibody and non-immune IgG (Santa Cruz, sc-2025) were used for ChIP. Primers used for ChIP-qPCR are as follows: forward primer: 5′- GCAAGTGGATCTCTGTGAAGTG -3′ and reverse primer: 5′- ATTCCTTAGGGA TCAGGGTGTT -3′.

### Statistical methods

All statistical analyses were performed using SPSS 21.0 (SPSS, USA) and GraphPad Prism 8 (GraphPad, USA) software. Student’s *t* test, one-way ANOVA (with Dunnett’s multiple comparison test) were used in the statistical analysis. Data in abnormal distribution were analyzed by non-parametric test. The Spearman non-parametric test was used to analyze correlations between *KLF6* relative mRNA levels and serum ALT/AST. The *χ*^2^ test was used for comparing KLF6 expression affected by clinical features of OLT patients. All experiments were repeated at least three times. In all figure legends, the information about the statistical details is indicated clearly. A *p* value <0.05 was considered statistically significant.

## Supplementary information

Reproducibility checklist

Table S2

Table S1

Figure S5

Figure S4

Figure S3

Figure S2

Figure S1

Supplementary Figure Legends

Original Data File

## Data Availability

The accession number for the RNA-Seq data reported in this paper is GSE216522 and is publicly accessible at https://www.ncbi.nlm.nih.gov/geo/. All data generated or analyzed during this study are included in this published article and its Supplementary files and available from the corresponding authors on request.
