# Supplementary figures and images for: KLF6 alleviates hepatic ischemia-reperfusion injury by inhibiting autophagy
Source: Cell Death Dis. 2023 Jul 1;14(7):393. doi: 10.1038/s41419-023-05872-3 (PMC10313896; doi:10.1038/s41419-023-05872-3)

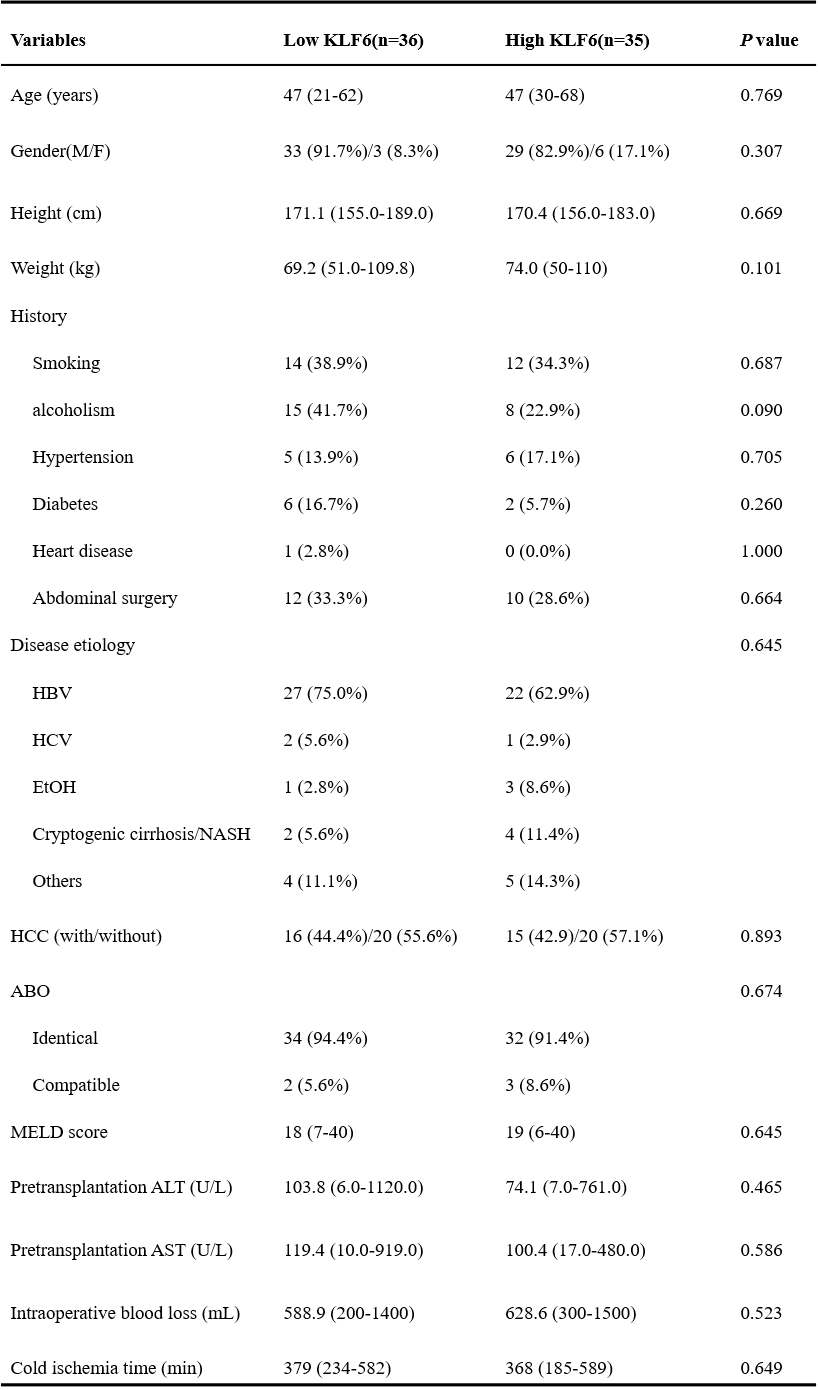


**Table S2. Recipients’ perioperative variables**

Supplement: Supplementary file 2 — Table S2 [file 41419_2023_5872_MOESM2_ESM.docx]

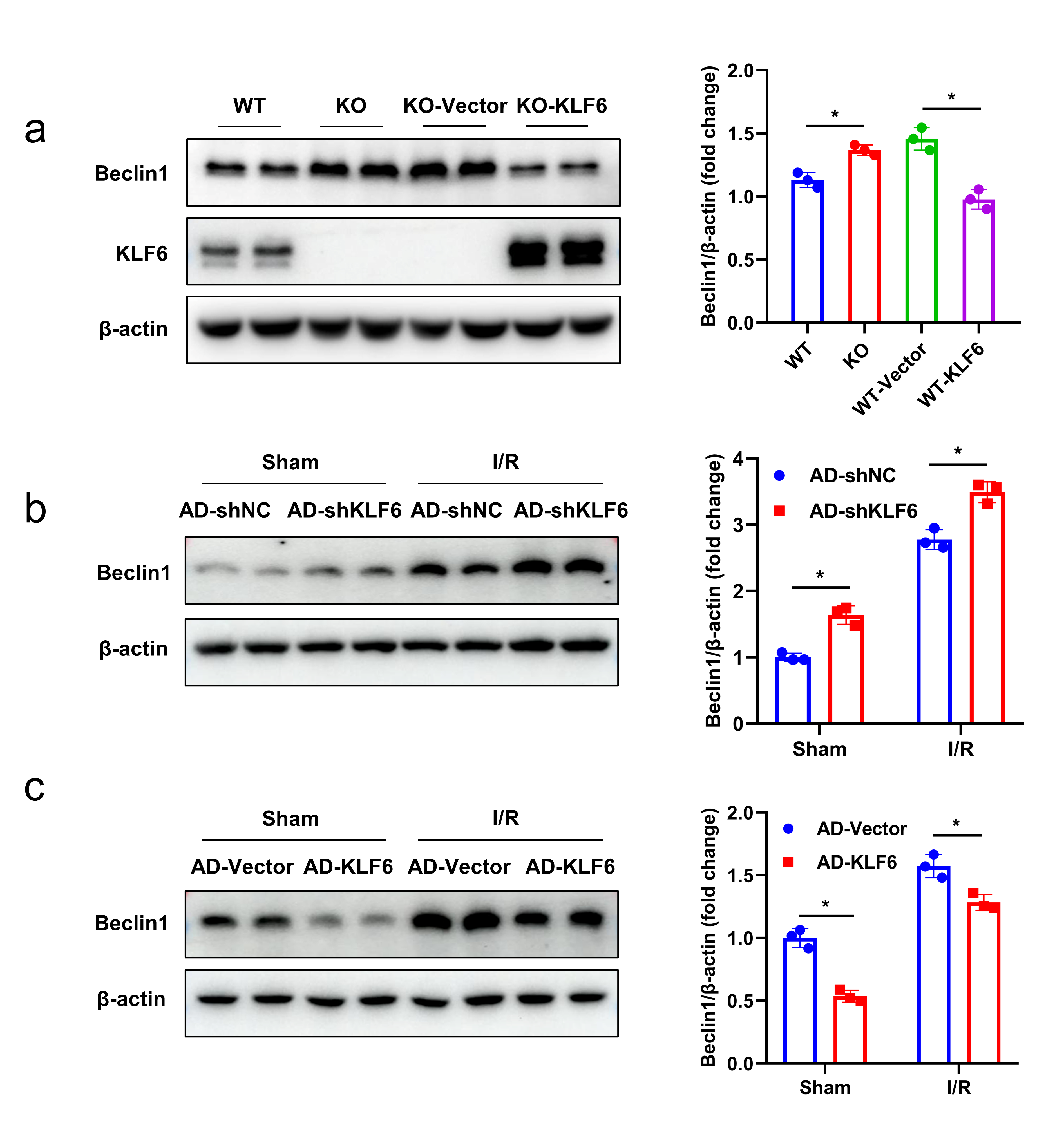

Supplement: Supplementary file 4 — Figure S5 [file 41419_2023_5872_MOESM4_ESM.tif]

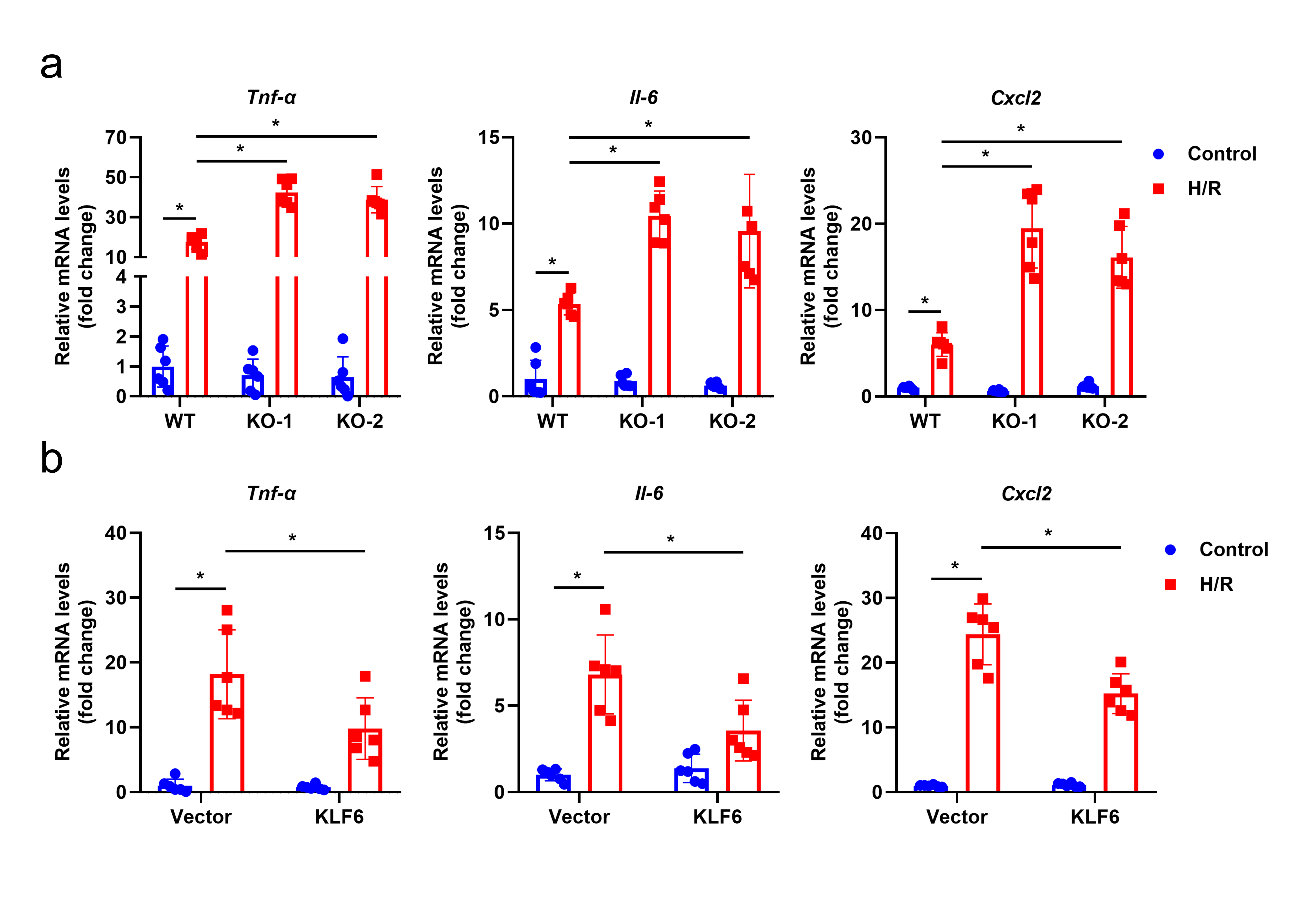

Supplement: Supplementary file 5 — Figure S4 [file 41419_2023_5872_MOESM5_ESM.tif]

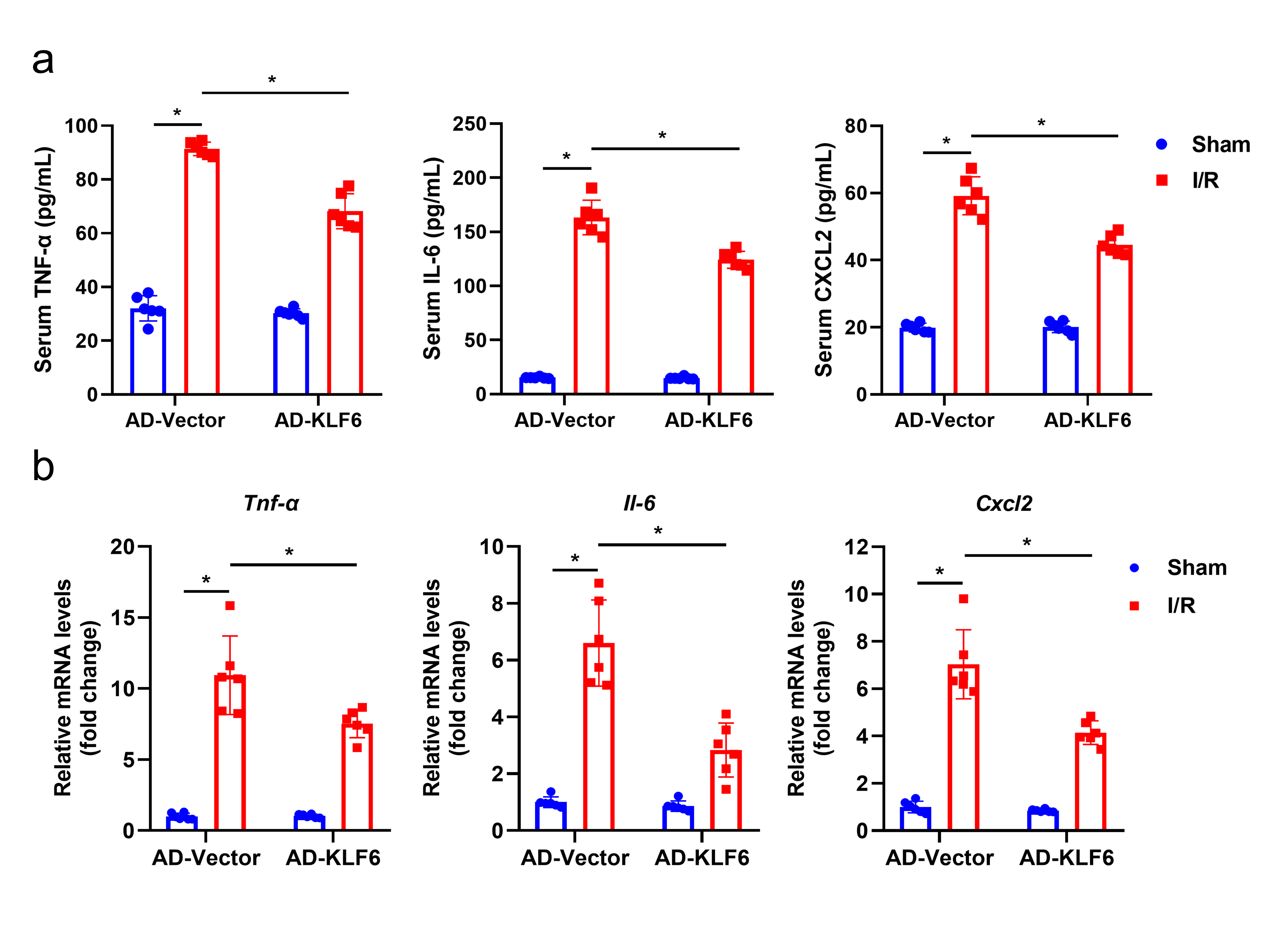

Supplement: Supplementary file 6 — Figure S3 [file 41419_2023_5872_MOESM6_ESM.tif]

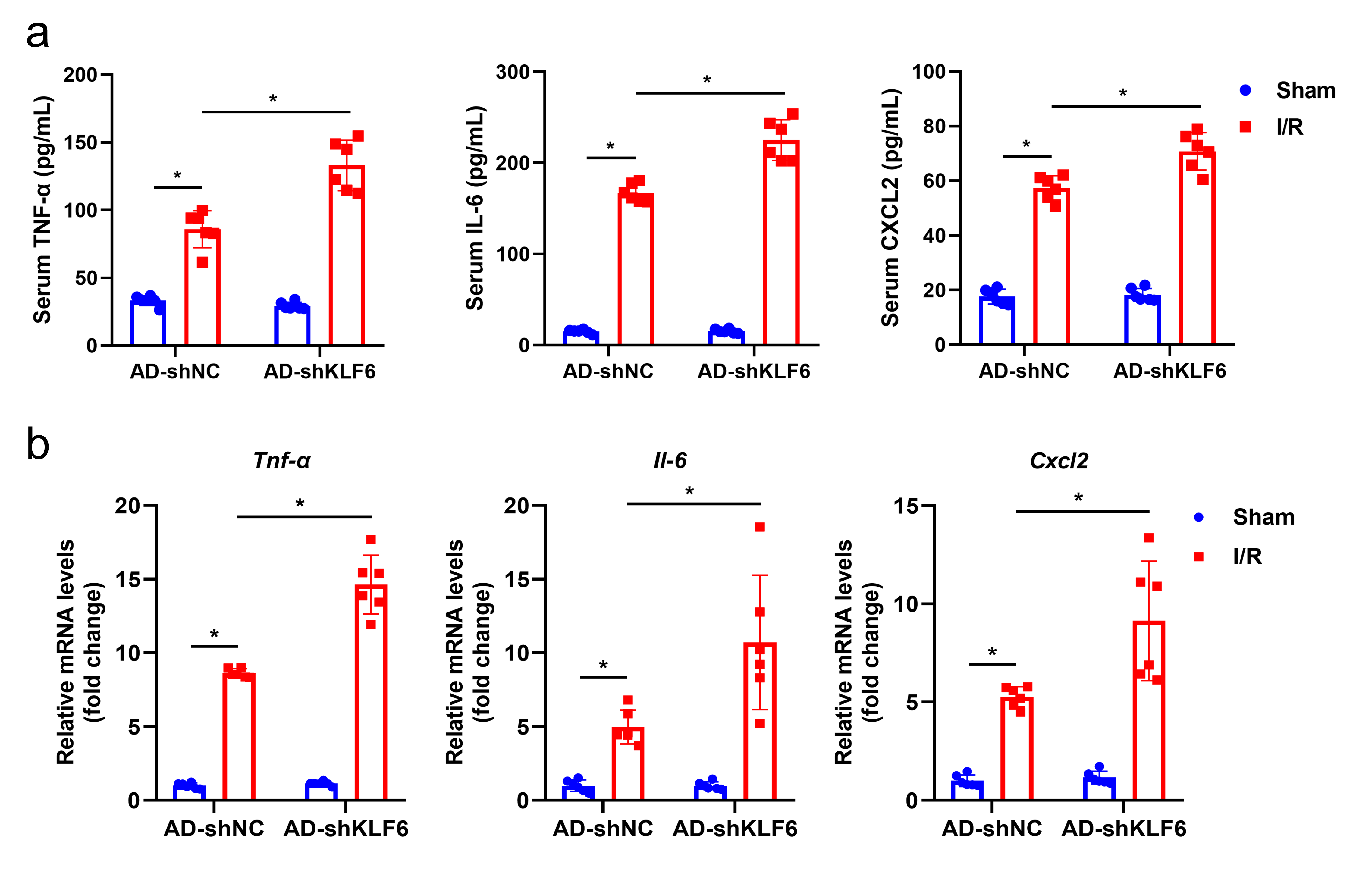

Supplement: Supplementary file 7 — Figure S2 [file 41419_2023_5872_MOESM7_ESM.tif]

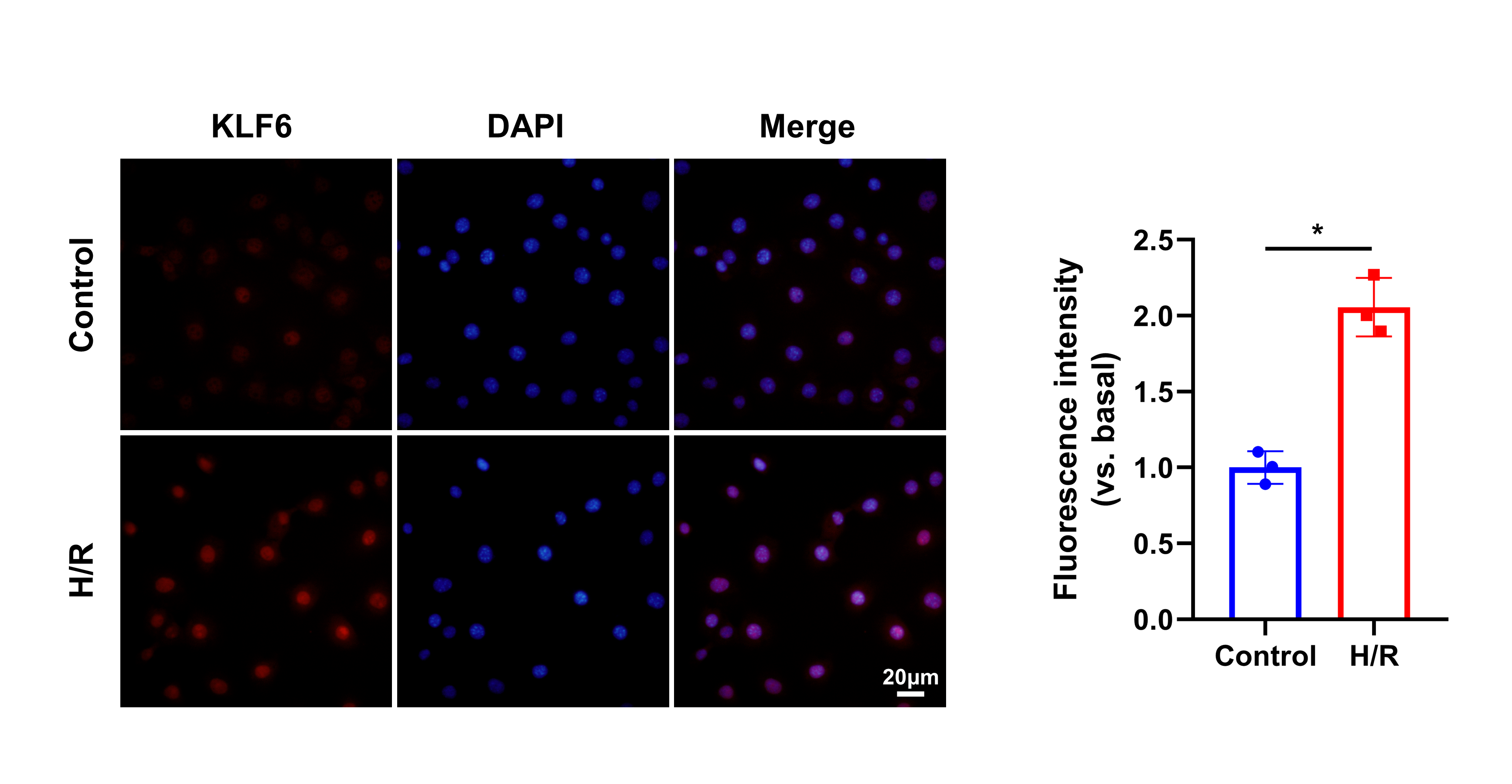

Supplement: Supplementary file 8 — Figure S1 [file 41419_2023_5872_MOESM8_ESM.tif]
