# Supplementary material for: KLF6 alleviates hepatic ischemia-reperfusion injury by inhibiting autophagy
Source: Cell Death Dis. 2023 Jul 1;14(7):393. doi: 10.1038/s41419-023-05872-3 (PMC10313896; doi:10.1038/s41419-023-05872-3)
Supplement: Supplementary file 3 — Table S1 [file 41419_2023_5872_MOESM3_ESM.docx]

| Gene | Sequence (5’-3’) | Species |
| --- | --- | --- |
| KLF6 forward | ACTGTCTTTTCCAACCCGAC | mouse |
| KLF6 reverse | AAGATAGCGTTCCAACTCCAG | mouse |
| β-actin forward | GGCTGTATTCCCCTCCATCG | mouse |
| β-actin reverse | CCAGTTGGTAACAATGCCATGT | mouse |
| TNF-α forward | TAGCCCACGTCGTAGCAAAC | mouse |
| TNF-α reverse | GCAGCCTTGTCCCTTGAAGA | mouse |
| IL-6 forward | AGAGACTTCCATCCAGTTGCC | mouse |
| IL-6 reverse | TCCTCTGTGAAGTCTCCTCTCC | mouse |
| CXCL2 forward | CCCAGACAGAAGTCATAGCCAC | mouse |
| CXCL2 reverse | TGGTTCTTCCGTTGAGGGAC | mouse |
| ATG13 forward | CCAGGCTCGACTTGGAGAAAA | mouse |
| ATG13 reverse | AGATTTCCACACACATAGATCGC | mouse |
| ATG14 forward | GAGGGCCTTTACGTGGCTG | mouse |
| ATG14 reverse | AATAGACGAAATCACCGCTCTG | mouse |
| ULK1 forward | GAGACCGTTGCTGACTCCAA | mouse |
| ULK1 reverse | TCCTAGAGAGAACAGGGGGC | mouse |
| Beclin1 forward | GAGGCATGGAGGGGTCTAAG | mouse |
| Beclin1 reverse | AGCTCGTGTCCAGTTTCAGAG | mouse |
| ATG3 forward | ACACGGTGAAGGGAAAGGC | mouse |
| ATG3 reverse | TGGTGGACTAAGTGATCTCCAG | mouse |
| ATG5 forward | TCACTGGCATAGCAAGGGTG | mouse |
| ATG5 reverse | ACAGTGACCAACGTAACAGCA | mouse |
| ATG7 forward | TGCCTCACCAGATCCGGGGTT | mouse |
| ATG7 reverse | CGCTAGGAAGGTGAATCCTTCTCG | mouse |
| KLF6 forward | CAAGGGAAATGGCGATGCCT | human |
| KLF6 reverse | CTTTTCTCCTGTGTGCGTCC | human |
| β-actin forward | ACCTTCTACAATGAGCTGCG | human |
| β-actin reverse | CCTGGATAGCAACGTACATGG | human |

**Table S1. Real time PCR primers.**
