# Supplementary material for: KLF6 alleviates hepatic ischemia-reperfusion injury by inhibiting autophagy
Source: Cell Death Dis. 2023 Jul 1;14(7):393. doi: 10.1038/s41419-023-05872-3 (PMC10313896; doi:10.1038/s41419-023-05872-3)
Supplement: Supplementary file 9 — Supplementary Figure Legends [file 41419_2023_5872_MOESM9_ESM.docx]

**Supplementary Figure Legends**

**Fig. S1 Immunofluorescence detected the KLF6 protein expression after H/R.** Scale bar, 20 μm. Mean values ± SD are shown, and statistical significance was determined by two-tailed Student’s t test. **P* < 0.05.

**Fig. S2 KLF6 knockdown exacerbates liver inflammation induced by I/R.** Liver tissue levels of inflammatory cytokines *Tnf-α*, *Il-6*, and *Cxcl2* were determined by ELISA (**a**) and RT-qPCR (**b**). Each group includes 6 mice. Mean values ± SD are shown, and statistical significance was determined by two-tailed Student’s t test. **P* < 0.05.

**Fig. S3 KLF6 overexpression ameliorates liver inflammation induced by I/R.** Liver tissue levels of inflammatory cytokines *Tnf-α*, *Il-6*, and *Cxcl2* were determined by ELISA (**a**) and RT-qPCR (**b**). Each group includes 6 mice. Mean values ± SD are shown, and statistical significance was determined by two-tailed Student’s t test. **P* < 0.05.

**Fig. S4 KLF6 inhibits** **inflammation in AML12 cells after H/R.** *Tnf-α, Il-6, Cxcl2* levels were determined after KLF6 knockout (**a**) or overexpression (**b**) in AML12 cells by RT-qPCR. Abbreviations: WT, wild type AML12 cells; KO, KLF6 knockout AML12 cells; Vector, AML12 cells were infected with an empty control lentiviral vector; KLF6, AML12 cells were infected with a lentiviral construct expressing KLF6. Mean values ± SD are shown, and statistical significance was determined by one-way ANOVA followed by Dunnett’s multiple comparison test (**a**) or two-tailed Student’s t test (**b**). **P* < 0.05.

**Fig. S5 KLF6 suppresses Beclin1 expression in vitro and in vivo. a** Western blot analysis of Beclin1 protein level after KLF6 knockout or overexpression in AML12 cells. **b, c** Western blot analysis of Beclin1 protein level after adenoviral mediated KLF6 knockdown or overexpression in mice (n=6). Abbreviations: WT, wild type AML12 cells; KO, KLF6 knockout AML12 cells; KO-Vector, KO cells were infected with an empty control lentiviral vector; KO-KLF6, KO cells were infected with KLF6 overexpression lentiviruses. Mean values ± SD are shown, and statistical significance was determined by two-tailed Student’s t test. **P* < 0.05.
