# Supplementary material for: KLF6 alleviates hepatic ischemia-reperfusion injury by inhibiting autophagy
Source: Cell Death Dis. 2023 Jul 1;14(7):393. doi: 10.1038/s41419-023-05872-3 (PMC10313896; doi:10.1038/s41419-023-05872-3)

Figure 1f

IB: KLF6

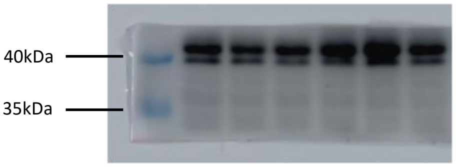

IB: β-actin

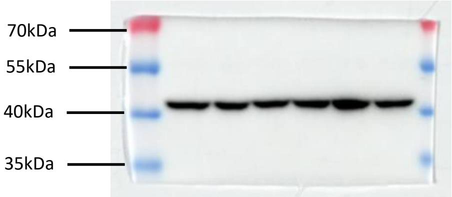

IB: KLF6

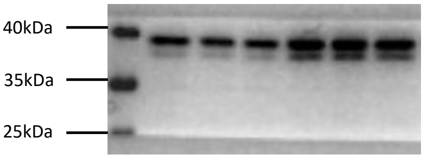

IB: β-actin

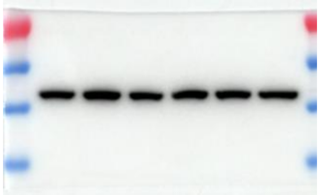

IB: KLF6

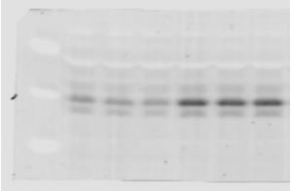

IB: β-actin

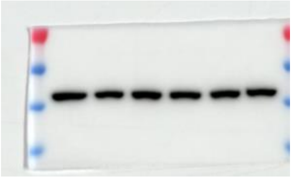

Figure 2b

IB: KLF6

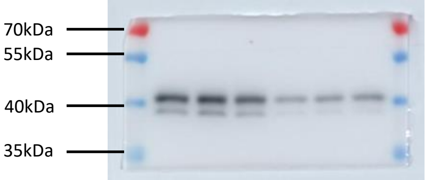

IB: β-actin

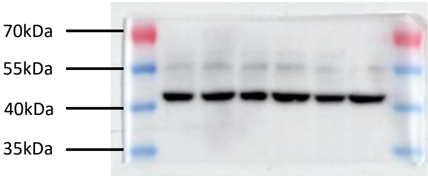

Figure 3b

IB: KLF6

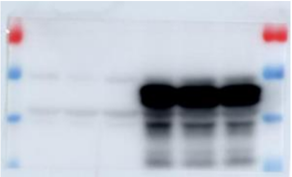

IB: β-actin

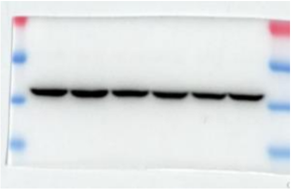

Figure 4a

IB: KLF6

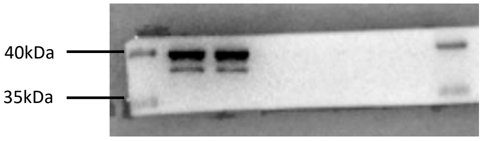

IB: β-actin

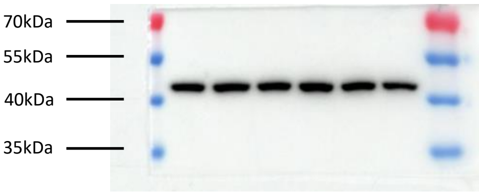

Figure 4c

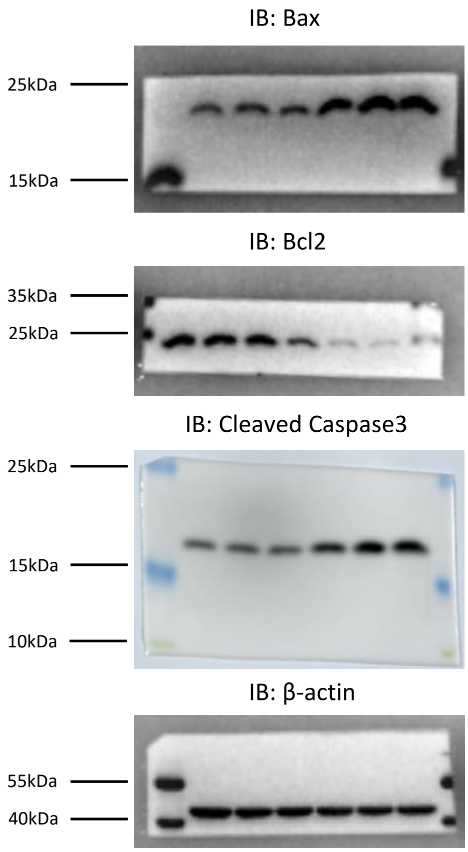

Figure 5b

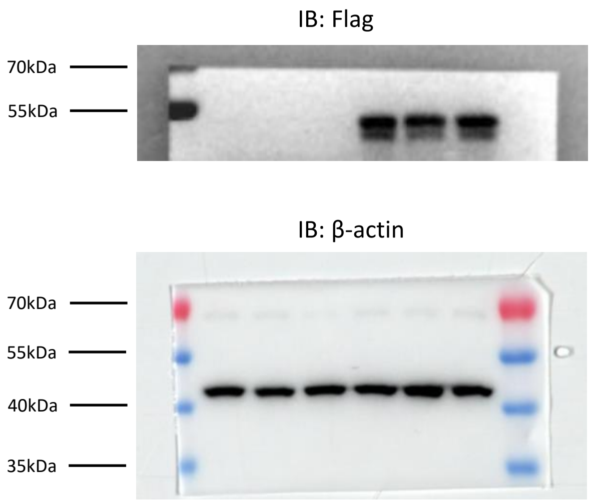

Figure 5d

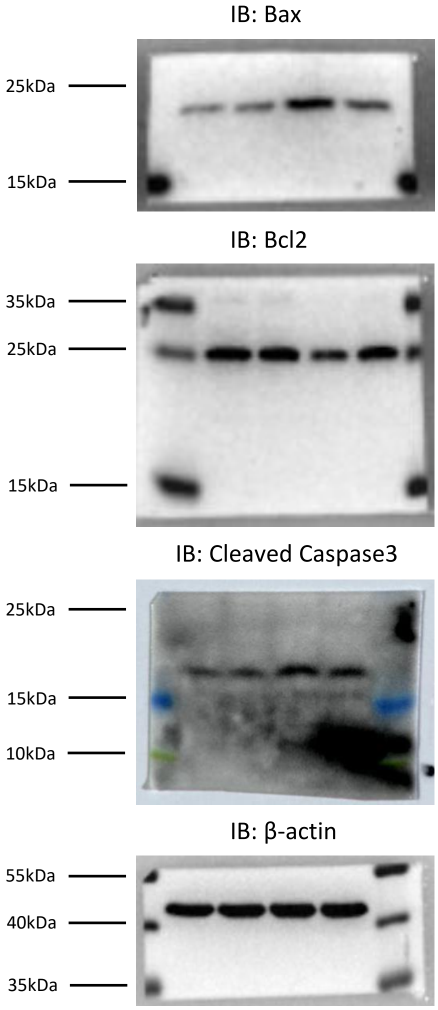

Figure 6a

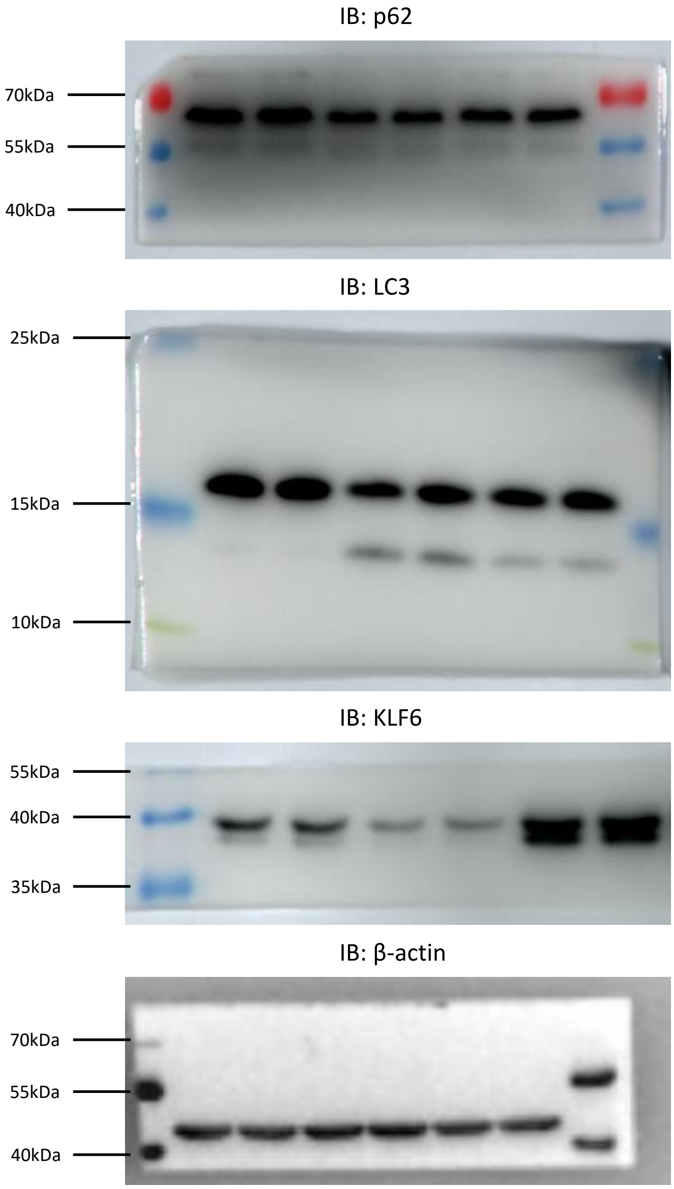
IB: p62
70kDa
55kDa
40kDa
IB: LC3
25kDa
15kDa
10kDa
IB: KLF6
55kDa
40kDa
35kDa
IB: β-actin
70kDa
55kDa
40kDa

Figure 6b

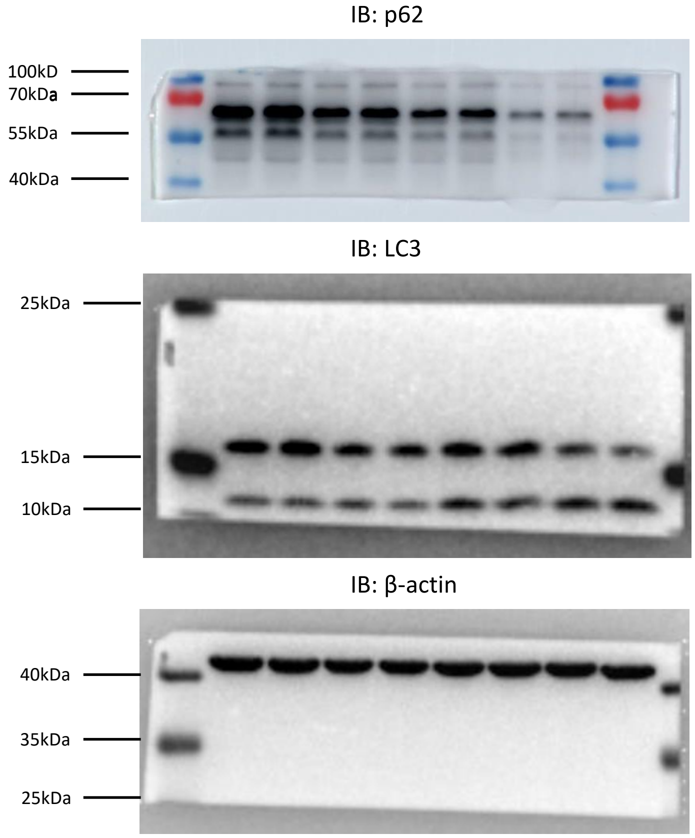

Figure 6c

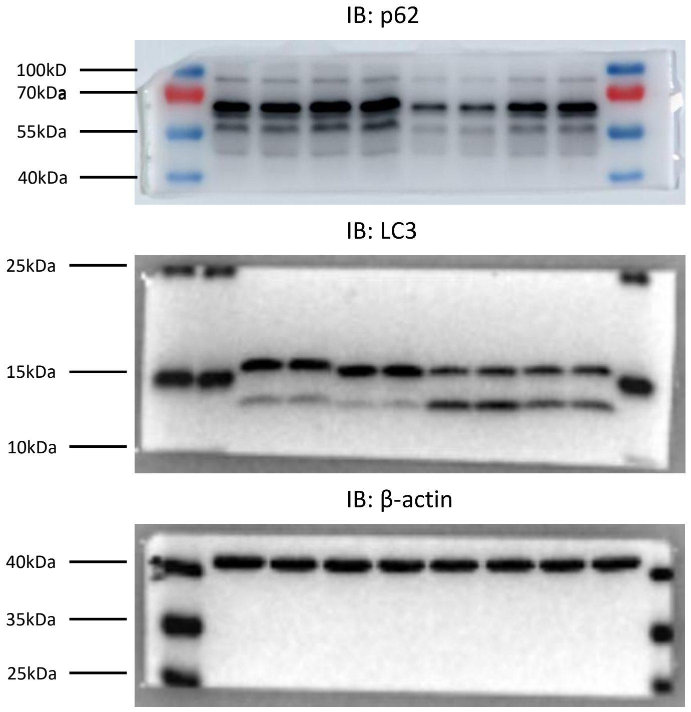

Figure 6d

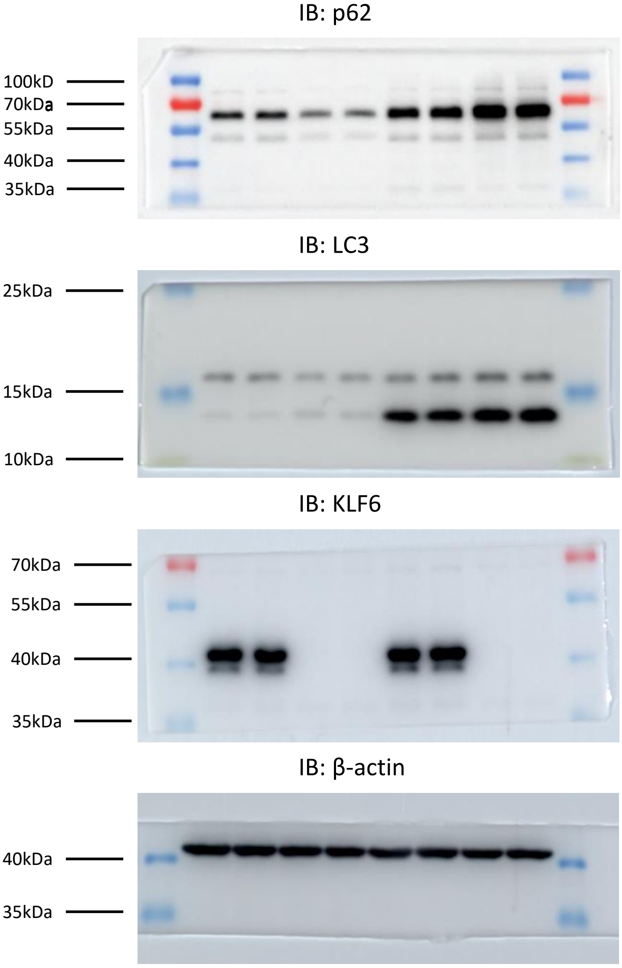

Figure 6e

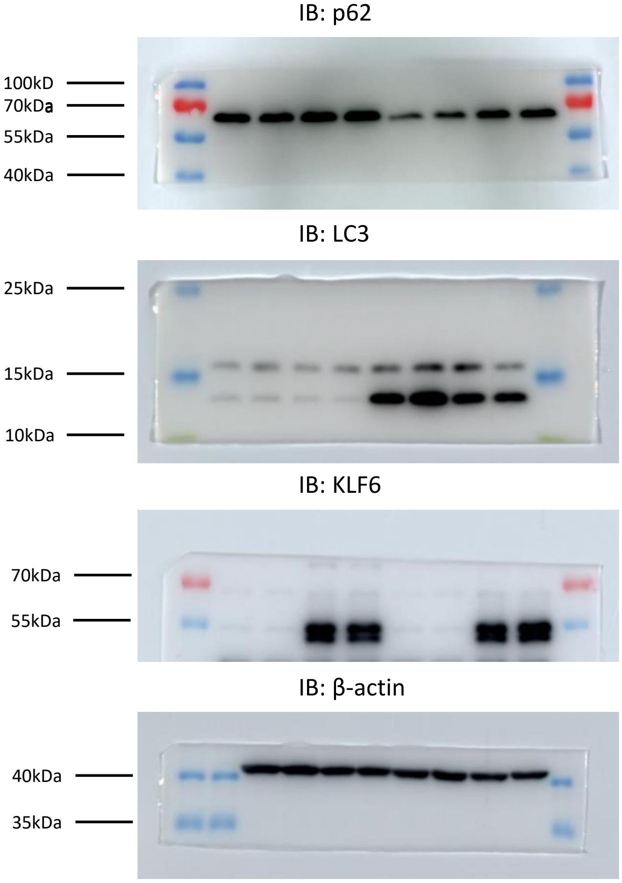

Figure 8b

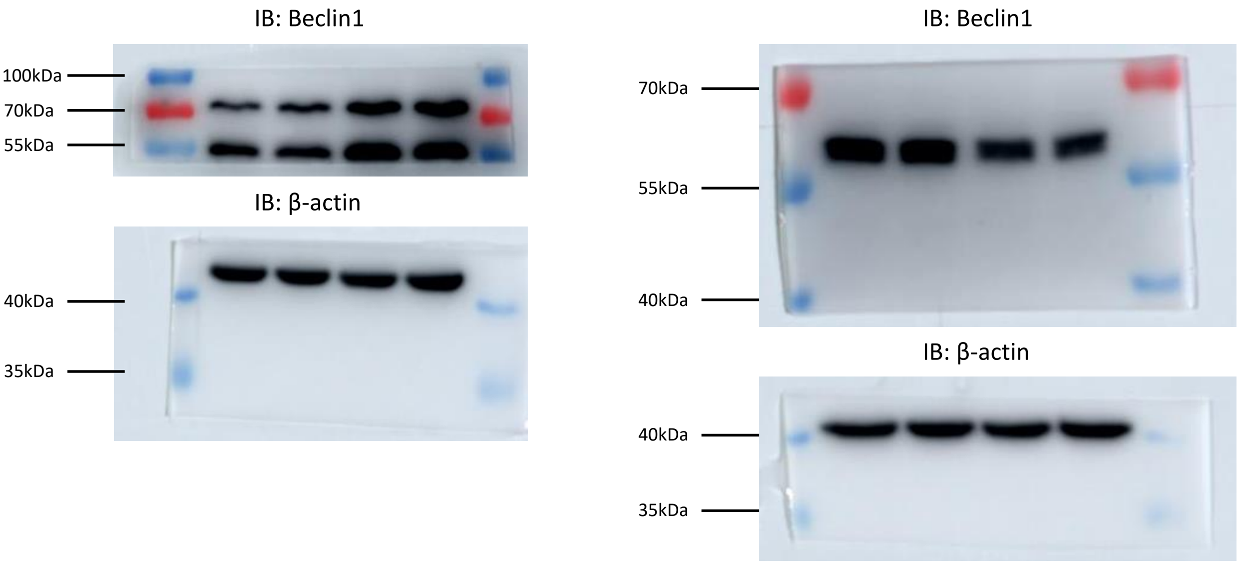

Figure S5a

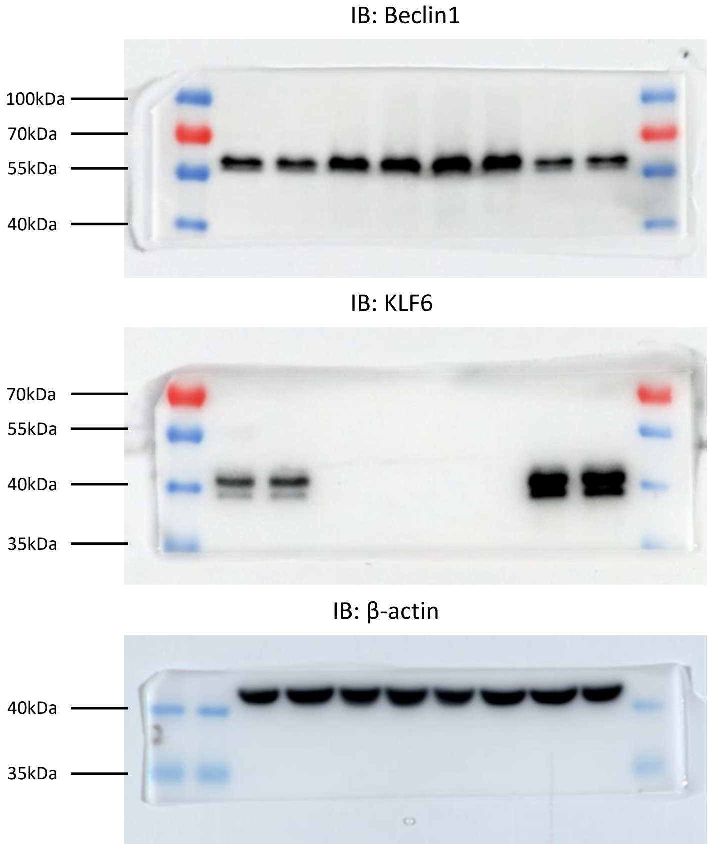

Figure S5b

IB: Beclin1

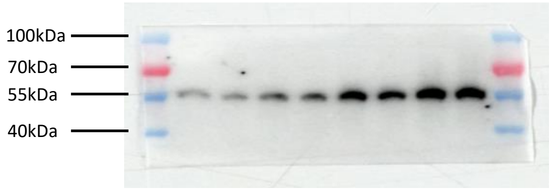

IB: β-actin

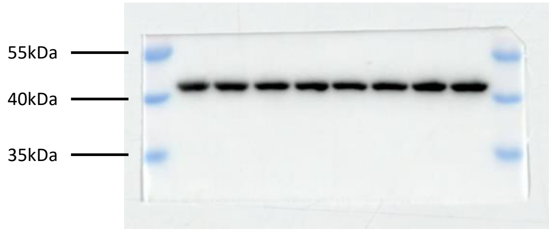

Figure S5c

IB: Beclin1

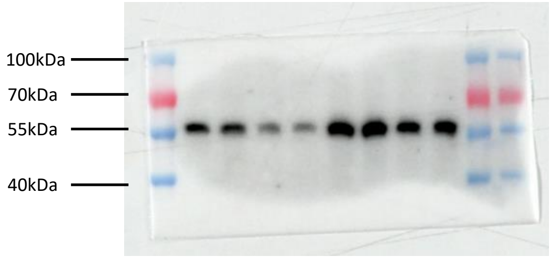

IB: β-actin

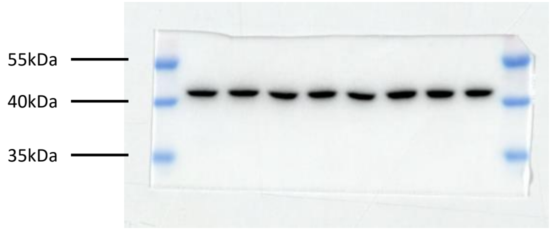

Figure 8e

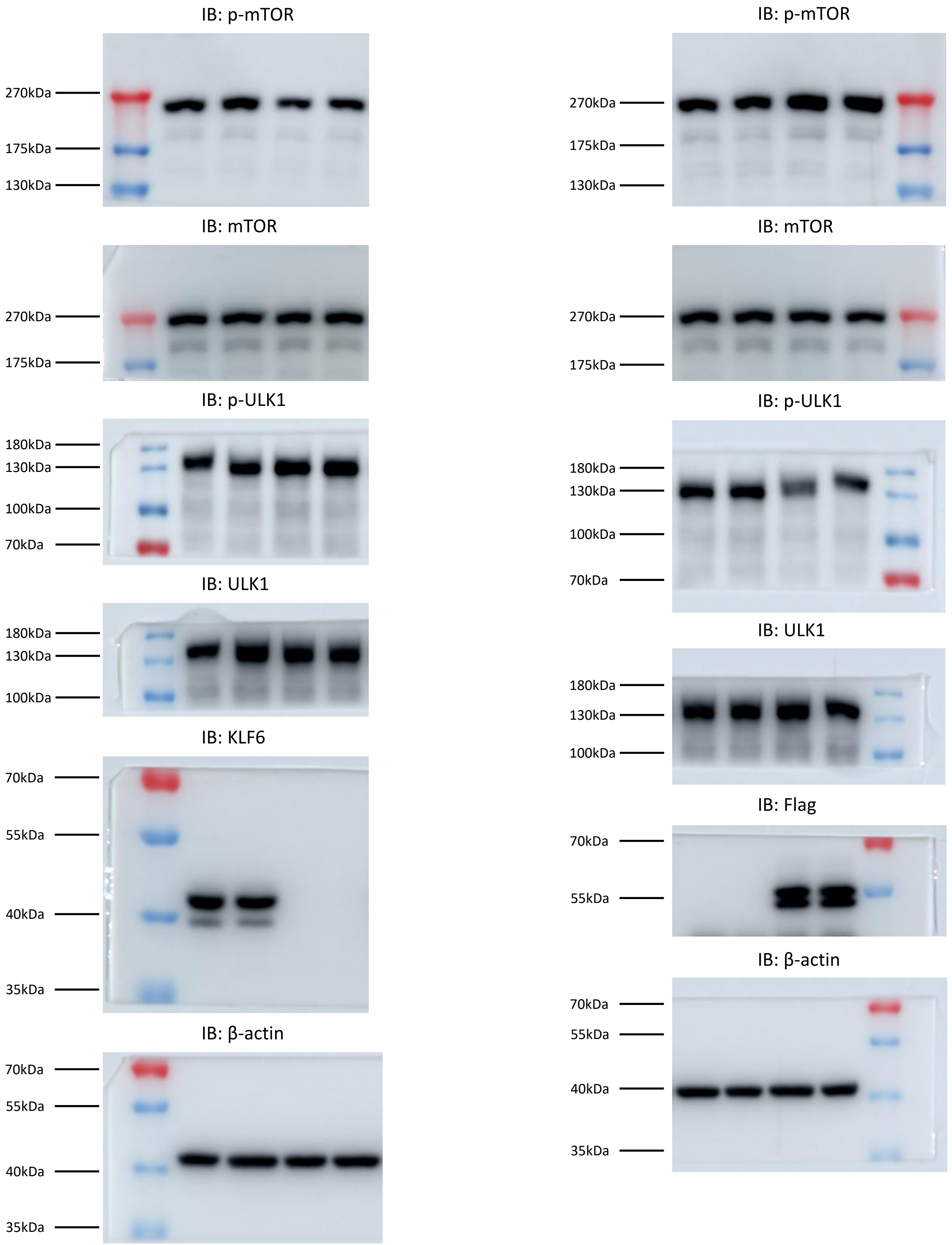
IB: p-mTOR
270kDa
175kDa
130kDa
IB: mTOR
270kDa
175kDa
IB: p-ULK1
180kDa
130kDa
100kDa
70kDa
IB: ULK1
180kDa
130kDa
100kDa
IB: KLF6
70kDa
55kDa
40kDa
35kDa
IB: β-actin
70kDa
55kDa
40kDa
35kDa
IB: p-mTOR
270kDa
175kDa
130kDa
IB: mTOR
270kDa
175kDa
IB: p-ULK1
180kDa
130kDa
100kDa
70kDa
IB: ULK1
180kDa
130kDa
100kDa
IB: Flag
70kDa
55kDa
IB: β-actin
70kDa
55kDa
40kDa
35kDa

Figure 9b

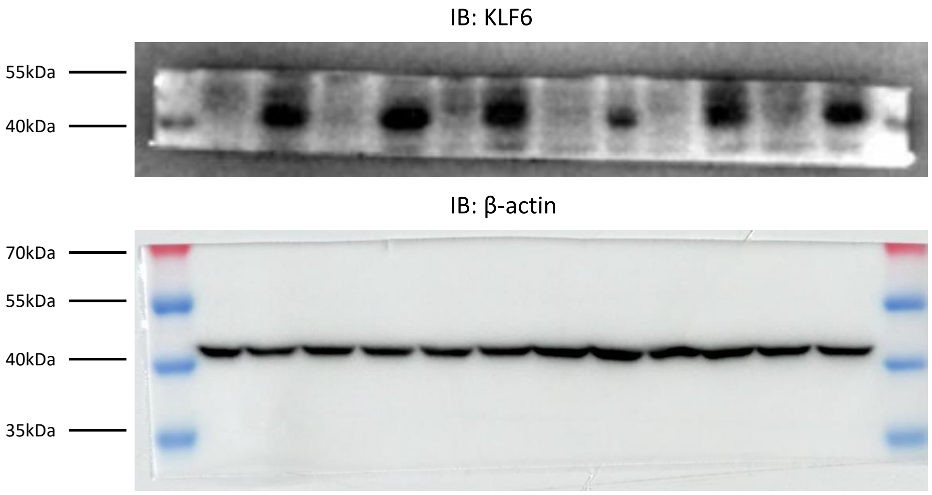

Supplement: Supplementary file 10 — Original Data File [file 41419_2023_5872_MOESM10_ESM.pdf]
